# Atomic‐Level Structural Characteristics of *β*‐Relaxation in Metallic Glasses

**DOI:** 10.1002/advs.202518424

**Published:** 2025-11-09

**Authors:** Tianding Xu, Jinquan Zhou, Xiao‐Dong Wang, Ke Yang, Qing‐Ping Cao, Dong‐Xian Zhang, Chih‐Wen Pao, Jian‐Zhong Jiang

**Affiliations:** ^1^ International Center for New‐Structured Materials (ICNSM) Laboratory of New‐Structured Materials Zhejiang University Hangzhou 310027 China; ^2^ State Key Laboratory of Silicon Materials and School of Materials Science and Engineering Zhejiang University Hangzhou 310027 China; ^3^ Shanghai Synchrotron Radiation Facility Zhangjiang Lab Shanghai Advanced Research Institute Chinese Academy of Science Shanghai 201210 China; ^4^ State Key Laboratory of Modern Optical Instrumentation Zhejiang University Hangzhou 310027 China; ^5^ National Synchrotron Radiation Research Center Hsin‐Ann Road, Hsinchu Science Park Hsinchu 30076 Taiwan; ^6^ Key Laboratory of Silicon‐based Materials The Ministry of Education Key Laboratory of Automotive Glass of Fujian Smart Automotive Glass Engineering Research Center of Fujian and School of Materials Science and Engineering Fuyao University of Science and Technology Fuzhou 350109 China

**Keywords:** β‐relaxation, chemical constitution, free volume, local structure, metallic glasses

## Abstract

The *β*‐relaxation is one of the crucial relaxation modes in glasses, which significantly influences their mechanical properties, structural heterogeneity, and glass transition. However, due to detection limitations, the *β*‐relaxation has not been fully characterized and understood. In this work, in situ synchrotron radiation X‐ray diffraction and absorption techniques are utilized to track the *β*‐relaxation during heating and find that it can be reflected in the evolution of cluster structures within the first shells of metallic glasses. Through molecular dynamics simulations, it is further demonstrated that the *β*‐relaxation is also well consistent with fast atomic motions linked with specific Voronoi polyhedra and chemical constitutions characterized by more excess free volume. The cyclic heating experiments provide evidence for the “irreversible” *β*‐relaxation, which involves an event that is decayed during the sub‐*T*_g_ annealing; however, it can be reactivated by subsequent high temperature rejuvenation. The discovery paves a new pathway for the study of *β*‐relaxation, unveiling the origin of *β*‐relaxation based on experimental and theoretical structural analysis methods.

## Graphical abstract

Using in situ synchrotron radiation X‐ray techniques, this study experimentally characterizes *β*‐relaxation at the atomic scale, linking it explicitly to tricapped trigonal prism (TTP)‐like atomic structures and fast atomic motions. Cyclic heating reveals *β*‐relaxation as irreversible yet reactivatable through rejuvenation, offering unprecedented insight into structural‐dynamic correlations essential for understanding metallic glasses’ macroscopic behaviors.

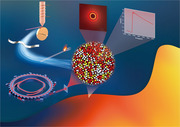

## 1 Introduction

*β*‐relaxation, one of the most prominent relaxation modes in glasses, is closely associated with physical aging, structural heterogeneity, and mechanical properties of glasses.^[^ ^1^, ^2^, ^3^, ^4^, ^5^, ^6^, ^7^, ^8^, ^9^, ^10^, ^11^, ^12^, ^13^ ^]^ Since its initial discovery in polymeric materials in the 1970s,^[^ ^14^, ^15^, ^16^ ^]^ *β*‐relaxation has attracted significant attention and is now recognized as a key factor in unraveling the mysteries of the glass transition. Metallic glasses (MGs), owing to their simple atomic structure and the absence of coordinated molecular motion and chain rotation, are often regarded as ideal model systems for studying *β*‐relaxation. As more MGs exhibiting pronounced *β*‐relaxation have been identified,^[^ ^17^, ^18^, ^19^, ^20^, ^21^, ^22^, ^23^, ^24^, ^25^ ^]^ intensive research has been devoted to the correlations between *β*‐relaxation and both mechanical properties and glass‐forming ability.

Yu et al.^[^ ^1^ ^]^ found that the activation energy for forming shear transformation zones (STZs), the primary carriers of plastic flow in MGs, is comparable to the activation energy for the *β*‐relaxation, both being about 26(±2)*RT*_g_ (*T*_g_: glass transition temperature). La‐based MGs with pronounced *β*‐relaxation were discovered to exhibit significant tensile plasticity, attributed to the onset of *β*‐relaxation slightly above room temperature.^[^ ^2^ ^]^ Subsequently, Wang^[^ ^22^ ^]^ and Küchemann et al.^[^ ^26^ ^]^ identified a *β*’‐relaxation (also called *γ* relaxation) at even lower temperatures, and found that it is closely related to the ductile‐brittle transition and low‐temperature plasticity. However, Atzmon et al.^[^ ^27^ ^]^ reported that the La_70_Cu_15_Al_15_ MG, which displays insignificant *β*‐relaxation, exhibited a more considerable tensile plasticity than the La_70_Ni_15_Al_15_ MG, which has significant *β*‐relaxation. Spieckermann et al.^[^ ^9^ ^]^ further suggested that the *γ*‐relaxation at low temperatures primarily triggers reversible deformation, whereas *β*‐relaxation is typically accompanied by irreversible structural rearrangements. Yang et al.^[^ ^28^ ^]^ proposed that the enhancement of *β*‐relaxation may be related to the formation of nanoglasses, resulting in improved plasticity. Through continuous heating experiments using flash differential scanning calorimetry (DSC), they observed a “shadow glass transition” in the heat flow curves, which might be correlated with *β*‐relaxation.^[^ ^17^ ^]^ In previous studies, Hu et al.^[^ ^29^, ^30^ ^]^ found that the enthalpy relaxation activation energy following the shadow glass transition is consistent with that of *β*‐relaxation, which was further supported by Zhu et al.^[^ ^7^ ^]^ via electron microscopy, demonstrating a correlation between *β*‐relaxation and both structural and spatial heterogeneity. Additionally, Zhao et al.^[^ ^31^ ^]^ observed the hidden sub‐*T*_g_ peaks (shadow glass transition) in DSC curves and proposed that *β*‐relaxation comprises both reversible and irreversible parts, corresponding to the sub‐*T*_g_ peak and enthalpy recovery, respectively. Consequently, despite considerable progress, *β*‐relaxation in MGs remains a subject of ongoing debate, and its underlying physical mechanisms and structural origins have not yet been fully clarified.

**Figure 1:**
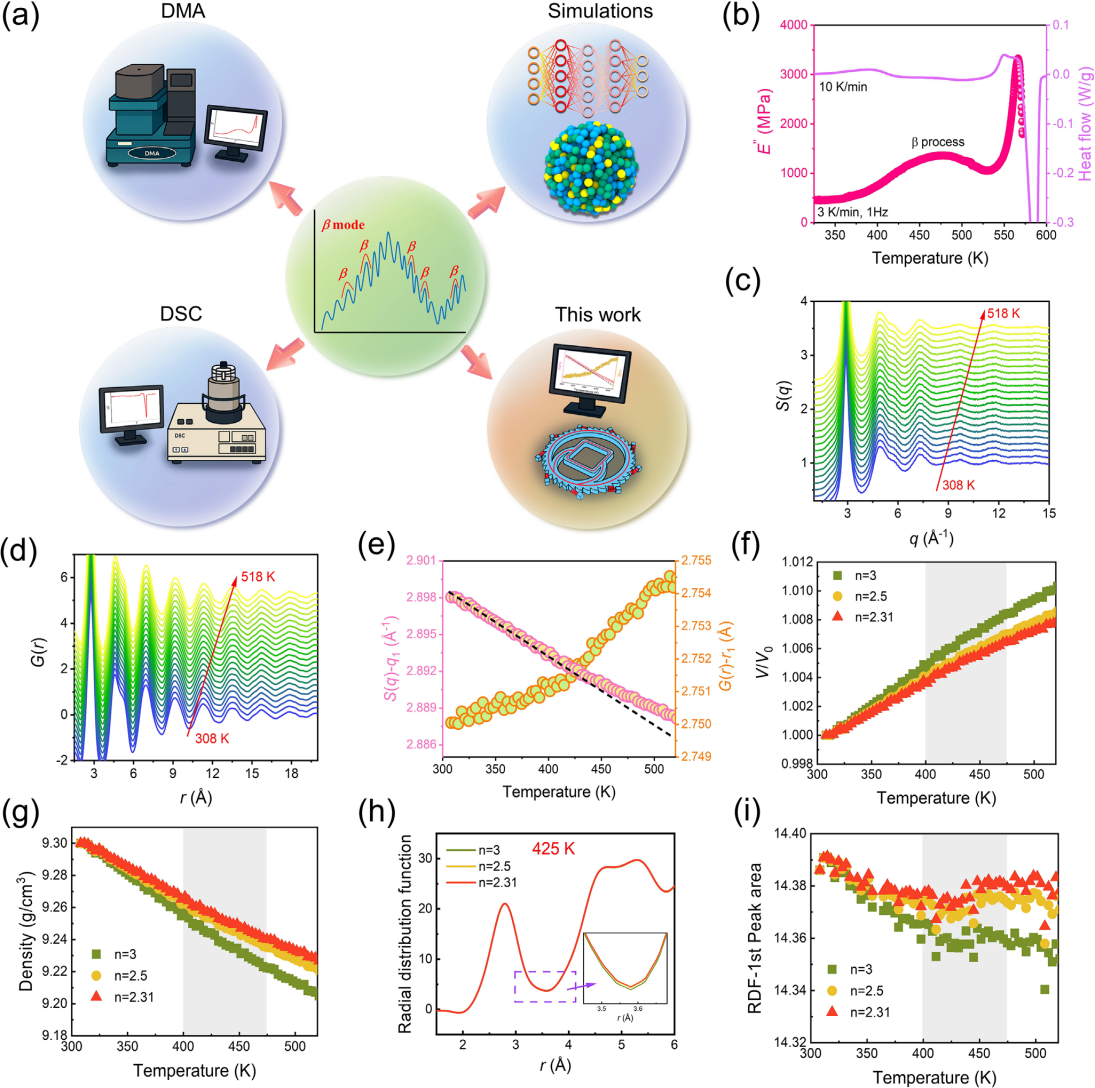
DMA/DSC profiles of Pd_40_Cu_40_P_20_ MG and the associated *β*‐relaxation are analyzed together with the thermal evolution of volume, density, radial distribution function, and coordination number under varying *n*. a) Principal research methods DMA, DSC, and MD on *β*‐relaxation in glassy materials over the past 25 years (light blue areas), and the SR‐based structural characterization approaches proposed in this work to capture *β*‐relaxation (the beige area). b) The temperature dependences of DMA loss modulus versus DSC heat flow. c) The static structure factors *S*(*q*) and d) pair distribution functions *G*(*r*) measured by HEXRD upon heating at 10 K min^−1^. e) The temperature dependences of the main peak position *q*_1_ of *S*(*q*) versus the corresponding main peak position *r*_1_ of *G*(*r*) upon heating. f) Evolution of the normalized volume with temperature upon heating under *n* = 3, 2.5, and 2.31, respectively. g) Corresponding densities at different temperatures, calculated based on volume and the sample's room‐temperature density. h) RDFs at 425 K extracted from HEXRD experiments with *n* = 3, 2.5, and 2.31, respectively, showing good overlapping. i) Temperature‐dependent changes in the nearest‐neighbor coordination number, obtained by integrating the first peak of the RDF.

Currently, most studies on the *β*‐relaxation in MGs rely on dynamic mechanical analysis (DMA) or on the heat‐flow transition signals obtained from DSC, with a scarcity of in situ structural characterizations. In this work, we present direct structural evidence of *β*‐relaxation obtained from in situ synchrotron radiation (SR) X‐ray techniques during heating (**Figure** **1**a), including high‐energy X‐ray diffraction (HEXRD) and X‐ray absorption fine structure spectroscopy (XAFS). Our findings reveal that: 1) the occurrence of *β*‐relaxation can be identified by tracking structural signatures in MGs during continuous heating; 2) *β*‐relaxation primarily originates from the evolution of specific atomic configurations centered on fast‐moving atoms; 3) the nature of *β*‐relaxation likely involves an event in which thermal activation is inhibited by irreversible free volume annihilation. These results not only demonstrate that the *β*‐relaxation entails irreversible atomic structural rearrangements but also provide unprecedented in situ experimental insights into its structural origins, thereby establishing a feasible approach for studying structural relaxation in other disordered materials.

## 2 Results

### 2.1 In situ X‐ray Diffraction

We select the Pd_40_Cu_40_P_20_ MG as a model system due to its pronounced *β*‐relaxation behavior.^[^ ^17^, ^25^ ^]^ Figure 1b presents the temperature dependence of heat flow and loss modulus for the Pd_40_Cu_40_P_20_ MG during continuous heating. A broad exothermic peak appearing between ≈400 and 530 K in the DSC curve is considered to be the thermodynamic signature of *β*‐relaxation,^[^ ^17^, ^31^ ^]^ which corresponds to a hump near 450 K in the loss modulus curve measured by DMA. To investigate the structural evolution associated with *β*‐relaxation, we employ in situ HEXRD to monitor structural changes during heating, obtaining both the structure factors *S*(*q*) and pair distribution functions *G*(*r*), as shown in Figure 1c,d. The primary peak positions of *S*(*q*) and *G*(*r*) are extracted to analyze their temperature dependencies, as illustrated in Figure 1e.

The position of the first peak, *q*_1_, in *S*(*q*) initially decreases linearly with temperature, from 2.898 to ≈2.892 Å^−1^, then exhibiting a marked deceleration after 425 K. This crossover is also detected in the temperature‐dependent first peak position *r*_1_ of *G*(*r*), showing a distinct kink in its increase with rising temperature. Additional evidence regarding the temperature evolution of other peak positions and peak heights in *S*(*q*) and *G*(*r*) is provided in Figures S1–S4 (Supporting Information). Interestingly, we observe that for other systems exhibiting weak or negligible *β*‐relaxation, such as CuZr_2_, Cu_46_Zr_46_Al_8_, and Pd_40_Ni_40_P_20_ MGs (see Figure S5a–f, Supporting Information), although complex relaxation dynamics may occur,^[^ ^25^, ^32^ ^]^ both *q*_1_ and *r*_1_ vary almost linearly with temperature and show noticeable changes only near *T*_g_. In contrast, for Cu_50_Zr_50_ and Cu_64_Zr_36_ MGs (Figure S5g–j, Supporting Information), which exhibit more pronounced *β*‐relaxation signals, clear transitions are already observable in the in situ HEXRD data in the glassy state, with both *q*_1_ and *r*_1_ shifting before *T*_g_, although less significantly than in Pd_40_Cu_40_P_20_ MG. These results demonstrate that in situ HEXRD can effectively probe the structural changes associated with *β*‐relaxation, revealing a structural crossover with the onset of *β*‐relaxation. To further understand the structural changes during the *β*‐relaxation in Pd_40_Cu_40_P_20_ MG, we perform the first‐principles molecular dynamics (FPMD) simulations using a heating‐relaxation‐heating protocol from 300 to 550 K. The first peak positions of *S*(*q*) are extracted and compared with the experimental data, as shown in Figure S6 (Supporting Information). The temperature dependence of the simulated *q*_1_ closely follows the experimental trend: as the temperature increases, *q*_1_ decreases monotonically from 2.885 Å^−1^ at 300 K, with a crossover near 425 K, almost capturing the experimental feature of a deviation from linearity ≈425 K. Using the main peak positions of the structure factors, we calculated the volume change of the system during heating through a simple power‐law relation, (2π/*q*_1_)*^n^* ∝V (see Figure 1f), where *n* represents the fractal dimension.^[^ ^33^, ^34^, ^35^, ^36^, ^37^, ^38^, ^39^, ^40^ ^]^ Regardless of the specific *n* values, we observe a crossover in the normalized volume of the system near 425 K (shaded area), with the volume increasing by ≈8% as the temperature approaches *T*_g_. Based on the volume change and the density of Pd_40_Cu_40_P_20_ MG at room temperature, we deduce the evolution of density during heating, as shown in Figure 1g, which decreases from 9.30 g cm^−3^ at room temperature to ≈9.22 g cm^−3^ near *T*_g_. The kinks observed ≈425 K further corroborate the occurrence of *β*‐relaxation, indicating a close relationship between *β*‐relaxation and density changes in the sample. Additionally, the area under the first peak in the radial distribution function (RDF) (see Figure 1h) associated with the first nearest neighbor coordination number (CN), shows a marked slowdown near 425 K, further supporting its relation to the onset of *β*‐relaxation, as shown in Figure 1i.

### 2.2 Local Structural Characterization

**Figure 2:**
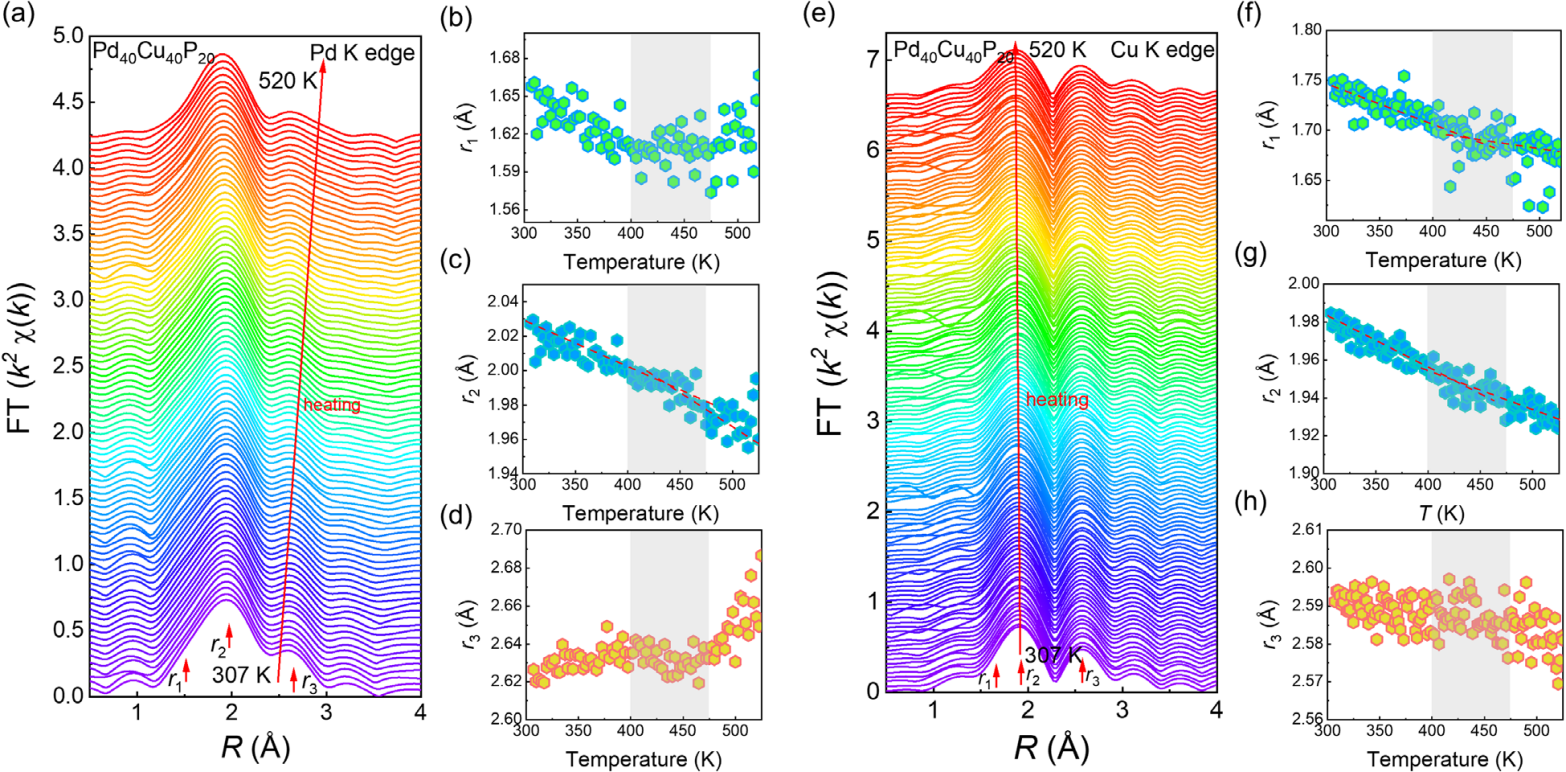
In situ high‐temperature XAFS peak evolution at the Pd and Cu K edges. a) The non‐phase‐shift corrected *R*‐space spectra of Pd *K*‐edge. b–d) Temperature‐dependent evolutions of the first peak position *r*_1_, the second peak position *r*_2,_ and the third peak position *r*_3_ derived from *R*‐space spectra of the Pd *K*‐edge. e) The non‐phase‐shift corrected *R*‐space spectra of Cu *K*‐edge. f–h) Temperature‐dependent evolutions of the first peak position *r*_1_, the second peak position *r*_2,_ and the third peak position *r*_3_ derived from *R*‐space spectra of the Cu *K*‐edge. The red dashed lines are guides for the eye.

XAFS is another powerful technique for probing local atomic structural changes around a selected element, such as CN and bond lengths. To further investigate the structural evolution, we conducted in situ high‐temperature XAFS experiments at both Cu and Pd *K* edges. **Figures** **2** and S7 (Supporting Information) present high‐quality Pd *K*‐edge and Cu *K*‐edge XAFS data in both *R*‐space and *k*‐space during the heating process of Pd_40_Cu_40_P_20_ MG. The *k* range extends up to 10 Å^−1^, ensuring high‐quality data in *R*‐space. The temperature‐dependent changes in peak positions (Figure 2b–d,f–h) exhibit kink‐like features ≈425 K, indicating that the atomic packing structures are closely associated with the occurrence of *β*‐relaxation. In Figure S8a (Supporting Information), the main peaks for Pd‐*K* and Cu *K*‐edge are attributed to Pd‐P/Cu and Cu‐Cu/Pd pairs, respectively, while the second peaks are mainly associated with Pd‐Pd and Cu‐Pd pairs. It is clear that the shoulder peak at 1.6 Å at the Pd *K*‐edge is more pronounced than that at the Cu *K*‐edge, indicating that P atoms are more likely to bond with Pd than with Cu. Notably, although the main peak intensities are comparable, the split‐peak intensities on the right side differ significantly, i.e., the peak intensity for Cu‐Pd pairs with long bond lengths at Cu *K*‐edge due to structural anharmonicity surpasses that of Pd‐Pd pairs at Pd *K*‐edge. This suggests that there is a greater prevalence of Cu─Pd bonds, which may provide more free volume around central Cu atoms. These experimental observations are quite consistent with the results from FPMD simulations in Figure S8b,c (Supporting Information), showing that Cu atoms surrounded by Pd atoms may have more available space, potentially leading to faster atomic dynamics.

### 2.3 Structural Changes During Thermal Cycling

**Figure 3:**
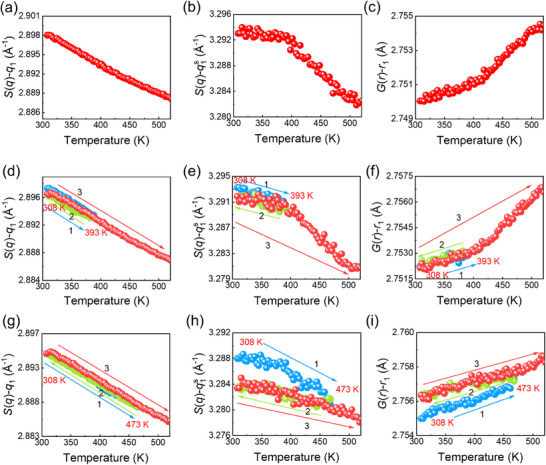
The structural evolution during cyclic heating. Temperature dependences of the main peak position *q*_1_, the shoulder peak position *q*_1_^s^ of *S*(*q*), and the main peak position *r*_1_ of *G*(*r*), respectively, measured by HEXRD upon a–c) one‐way continuous heating to 523 K; d–f) Thermal cycling where the sample is first heated from 308 to 393 K, cooled to 308 K, and then reheated to 523 K; and g–i) Similar thermal cycling processes, but with the first stop at 473 K. The heating and cooling rates for all steps are 10 K min^−1^.

The reversibility of *β*‐relaxation has been a topic of debate in recent years.^[^ ^9^, ^31^ ^]^ Here, we address this issue from a structural perspective by using cyclic heating experiments. First, **Figure** **3**a–c presents the results of a single heating protocol for the Pd_40_Cu_40_P_20_ MG, where the sample was heated from 308 K to near *T*_g_. A clear structural evolution is observed near 425 K, which is related to the *β*‐relaxation. Second, an as‐cast sample was heated to 393 K, just below the *β*‐relaxation onset, then cooled to room temperature and finally reheated to 523 K at a heating/cooling rate of 10 K min^−1^, as shown in Figure 3d–f. Despite undergoing the initial heating stage, the temperature dependence of the peak positions in *S*(*q*) and *G*(*r*) remains almost unchanged compared to the single heating protocol, exhibiting similar profiles. This suggests that cyclic heating and cooling below 393 K has a negligible effect on the *β*‐relaxation. Third, another as‐cast sample was heated to 473 K, above the *β*‐relaxation regime, cooled to 308 K, and reheated to 523 K at 10 K min^−1^, as shown in Figure 3g–i. Although the first heating to 473 K preserves the general structural transition (see blue solid spheres), the second heating completely removes the transition signal, showing a linear variation in both *q*_1_ and *r*_1_. These results reveal that heating above the *β*‐relaxation regime erases the irreversible structural signatures associated with *β*‐relaxation.

## 3 Discussion

### 3.1 Relationship Between β‐relaxation and Environments of Fast‐Moving Atoms

To elucidate the structural origins of pronounced *β*‐relaxation in MGs, we perform first‐principles molecular dynamics simulations for three MGs with pronounced *β*‐relaxation (La_50_Al_15_Ni_35_, Pd_40_Cu_40_P_20_, and Fe_86_Zr_8_B_6_) and two MGs without pronounced *β*‐relaxation (La_50_Al_15_Cu_35_ and Pd_40_Ni_40_P_20_) as references for comparison. In the latter two MGs, the *β*‐relaxation signal is submerged within the tail of *α*‐relaxation, as shown in Figure S9a (Supporting Information). The simulated pair distribution functions *G*(*r*) and structure factors *S*(*q*) for all five MGs are in good agreement with experimental data obtained via HEXRD measurements at 300 K (Figure S9b,c, Supporting Information).

**Figure 4:**
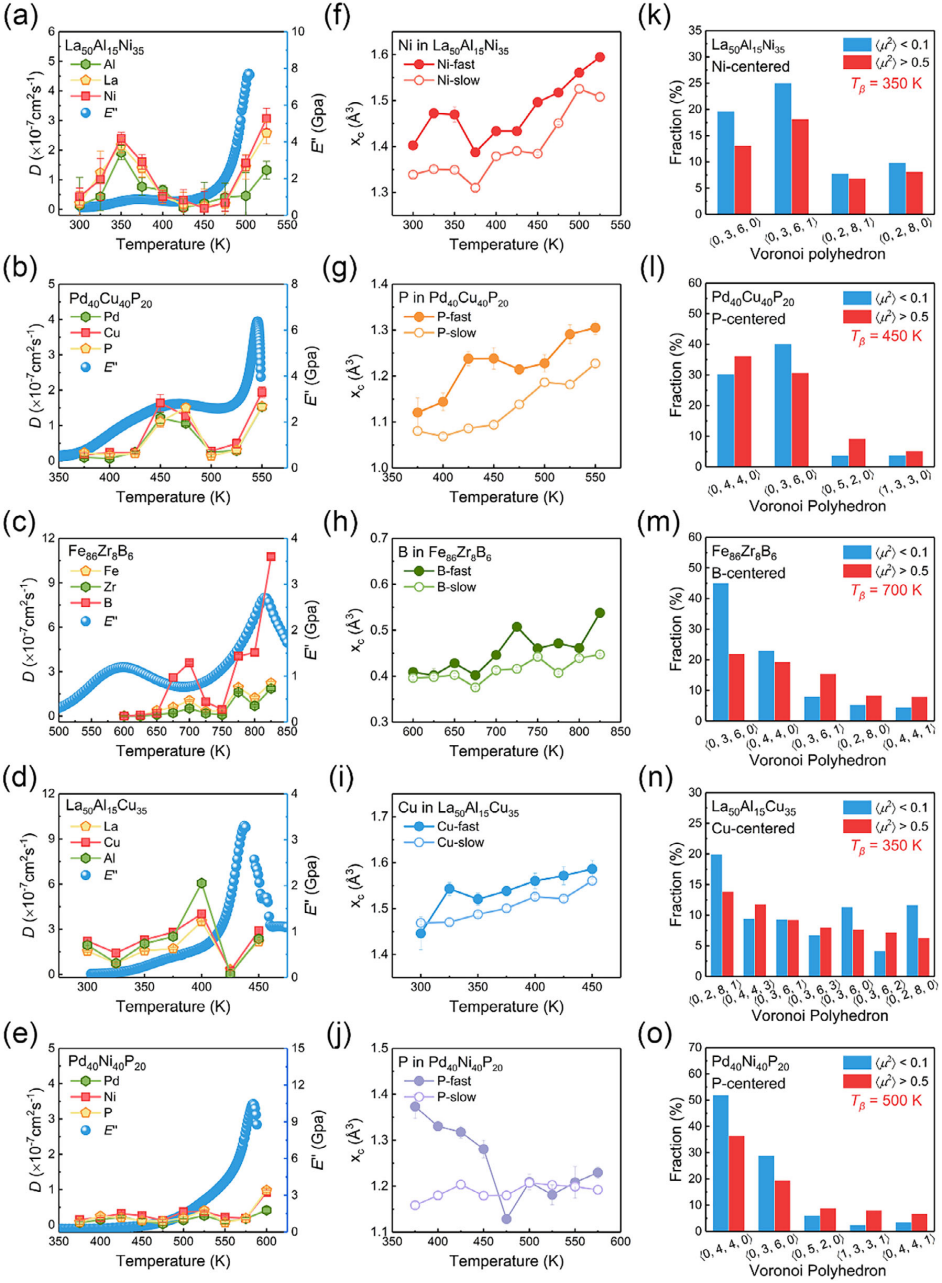
First‐principles calculations of the atomic diffusivity and static structure. a–e) Relationship between the self‐diffusion coefficient (*D*) and loss modulus (${E^{\prime }}^{\prime }$). The error bar indicates the standard error of the slope of the fitted line for the MSD curve. f–j) The fitted peak positions (*x*_c_) of the free volume distribution. The error bar represents the standard error of the *x*_c_ from the Gumbel fit. k–o) Fractions of major Voronoi polyhedra centered by smallest atoms (fast (〈*μ* ^2^〉 > 0.5 Å^2^) and slow (〈*μ* ^2^〉 < 0.1 Å^2^)) at *T_β_* of their own in La_50_Al_15_Ni_35_, Pd_40_Cu_40_P_20_, Fe_86_Zr_8_B_6_, La_50_Al_15_Cu_35_, and Pd_40_Ni_40_P_20_ MGs.

**Figure** **4**a–e presents the atomic diffusivity of the studied MGs, while their corresponding mean‐squared displacements (MSDs) are shown in Figure S10a–e (Supporting Information). The self‐diffusion coefficients (*D*) of all three constituent elements in La_50_Al_15_Ni_35_ MG (Figure 4a) exhibit a local maximum ≈350 K, followed by a gradual decrease until ≈450 K before increasing again at higher temperatures. Notably, the observed diffusivity hump near 350 K correlates well with the intensity variation of structural parameters ≈375 K, reflecting the *β*‐relaxation event. A similar dynamic slowdown has been previously reported for another La‐Al‐Ni MG at ≈363 K.^[^ ^41^ ^]^ Interestingly, analogous diffusivity trends are also observed for P atoms in Pd_40_Cu_40_P_20_ (Figure 4b) and for B atoms in Fe_86_Zr_8_B_6_ MG (Figure 4c). The above discussion focuses on ternary alloys; a natural question is whether similar phenomena also occur in the more common binary MGs. Here, we take the widely studied Cu‐Zr‐based MGs for further investigation. Consistent with the above observations, Cu_50_Zr_50_ and Cu_64_Zr_36_ MGs (Figure S11a,b, Supporting Information) also exhibit a diffusivity hump within the 550–650 K range, aligning precisely with their DMA‐detected *β*‐relaxation peaks. In contrast, MGs without pronounced *β*‐relaxation—including La_50_Al_15_Cu_35_ (Figure 4d), Pd_40_Ni_40_P_20_ (Figure 4e), as well as newly included CuZr_2_ and Cu_46_Zr_46_Al_8_ MGs (Figure S12a,b, Supporting Information)—show a steady increase in *D* with temperature and lack any noticeable humps within the *β*‐relaxation temperature ranges. To further quantify these differences, Figure S13 (Supporting Information) plots the normalized change in diffusivity (Δ*D* = *D_i_* – *D*_0_, where *D_i_* is the diffusivity at temperature *i* and *D*_0_ is the baseline value at initial temperature) for all systems. Δ*D* exhibits clear humps in MGs with pronounced *β*‐relaxation (La_50_Al_15_Ni_35_, Pd_40_Cu_40_P_20_, Fe_86_Zr_8_B_6_, Cu_50_Zr_50_, Cu_64_Zr_36_), while those with negligible *β*‐relaxation (La_50_Al_15_Cu_35_, Pd_40_Ni_40_P_20_, CuZr_2_, Cu_46_Zr_46_Al_8_) show monotonic increases in Δ*D* without humps. Notably, the magnitude of the Δ*D* change in La_50_Al_15_Ni_35_, Pd_40_Cu_40_P_20_, and Fe_86_Zr_8_B_6_ is larger than that in Cu_50_Zr_50_, Cu_64_Zr_36_, consistent with their correspondingly stronger *β*‐relaxation signals. We also analyze the fractions of “fast” atoms (defined as having Debye‐Waller factors 〈*μ* ^2^〉 > 0.5 Å^2^) and “slow” atoms (〈*μ* ^2^〉 < 0.1 Å^2^) in all five MGs (Figure S14, Supporting Information). The MGs exhibiting pronounced *β*‐relaxation contain significantly higher fractions of fast atoms than those without pronounced *β*‐relaxation, indicating that pronounced *β*‐relaxation could be closely related to the behaviors of these fast‐moving atoms.

The free volume distributions of the smallest atomic species in all five MGs were calculated and fitted using the Gumbel function (see details in Figures S15–S21, Supporting Information). In systems exhibiting pronounced *β*‐relaxation, the fitted peak positions (*x*_c_) of free volume distributions for fast atoms—such as Ni in La_50_Al_15_Ni_35_, P in Pd_40_Cu_40_P_20_, and B in Fe_86_Zr_8_B_6_—display a distinct local hump around their respective relaxation temperatures (e.g., near 350 K for La_50_Al_15_Ni_35_, as shown in Figure 4f–h), consistent with the anomalous diffusivity behavior observed in these systems. Similarly, fast Cu atoms in Cu_50_Zr_50_ and Cu_64_Zr_36_ MGs (Figure S11c,d, Supporting Information) also exhibit an *x*_c_ hump between 550 and 650 K, aligning with their diffusivity hump. In contrast, no such humps are observed in *x*_c_ values for slow or total atoms in any of these MGs (Figures S11c,d and S22a–c, Supporting Information), nor in any atomic subgroups (fast, slow, or total) of systems lacking pronounced *β*‐relaxation, including La_50_Al_15_Cu_35_ (Figure 4i; Figure S23a, Supporting Information), Pd_40_Ni_40_P_20_ (Figure 4j; Figure S23b, Supporting Information), CuZr_2_ and Cu_46_Zr_46_Al_8_ MGs (Figure S12c,d, Supporting Information). Notably, in Pd_40_Ni_40_P_20_, the *x*_c_ value for fast P atoms even decreases anomalously with increasing temperature. Figures S11c,d, S12c,d, S22a–c and S23a,b (Supporting Information) reveal that the free volume values of the overall atoms in all nine systems, similar to those of slow atoms, do not exhibit the characteristic hump observed in fast atoms from systems with distinct *β*‐relaxation. These results strongly indicate that only an increase in free volume around fast‐moving atoms is conducive to *β*‐relaxation.

The local atomic configurations were analyzed using Voronoi polyhedral analyses.^[^ ^42^, ^43^ ^]^ In the La_50_Al_15_Ni_35_ MG, the dominant Ni‐centered polyhedra are 〈0, 3, 6, 0〉 and 〈0, 3, 6, 1〉 (Figure 4k), while Cu‐centered Voronoi polyhedra (VP) are mainly 〈0, 2, 8, 1〉 in the La_50_Al_15_Cu_35_ MG (Figure 4n). For Pd_40_Cu_40_P_20_ and Fe_86_Zr_8_B_6_ MGs, the dominant VP centered by P and B atoms are 〈0, 3, 6, 0〉 and 〈0, 4, 4, 0〉 types, respectively (Figure 4l,m). Both are tricapped trigonal prism (TTP)‐like structures associated with relatively excess free volume. However, the Pd_40_Ni_40_P_20_ MG also predominantly contains TTP‐like polyhedra centered by P atoms (Figure 4o) yet exhibits insignificant *β*‐relaxation. It implies that TTP‐like polyhedra may constitute a necessary but insufficient structural condition for pronounced *β*‐relaxation. Hence, additional factors, such as specific chemical environments, likely influence atomic dynamics and relaxation behavior (more details can refer to Figure S24, Supporting Information). In the Cu‐Zr‐based MGs, Voronoi analysis reveals that Cu‐centered polyhedra are predominantly icosahedron‐like (e.g., 〈0, 0,12, 0〉, 〈0, 2, 8, 1〉, 〈0, 2, 8, 2〉) rather than TTP‐type structures.^[^ ^44^, ^45^ ^]^ In addition to the marked differences in free volume between the overall and fast atoms, distinct variations in VP distributions are observed between the total and fast atoms across all nine studied systems (Figures S11e,f, S12e,f, S22d–f and S23c,d, Supporting Information). These results indicate that the overall structural distribution does not accurately represent the static configuration of fast atoms. On the contrary, it is the fast atoms rather than the overall atomic arrangement in Pd_40_Cu_40_P_20_, La_50_Al_15_Ni_35_, and Fe_86_Zr_8_B_6_ that are closely associated with the significant *β*‐relaxation.

**Figure 5:**
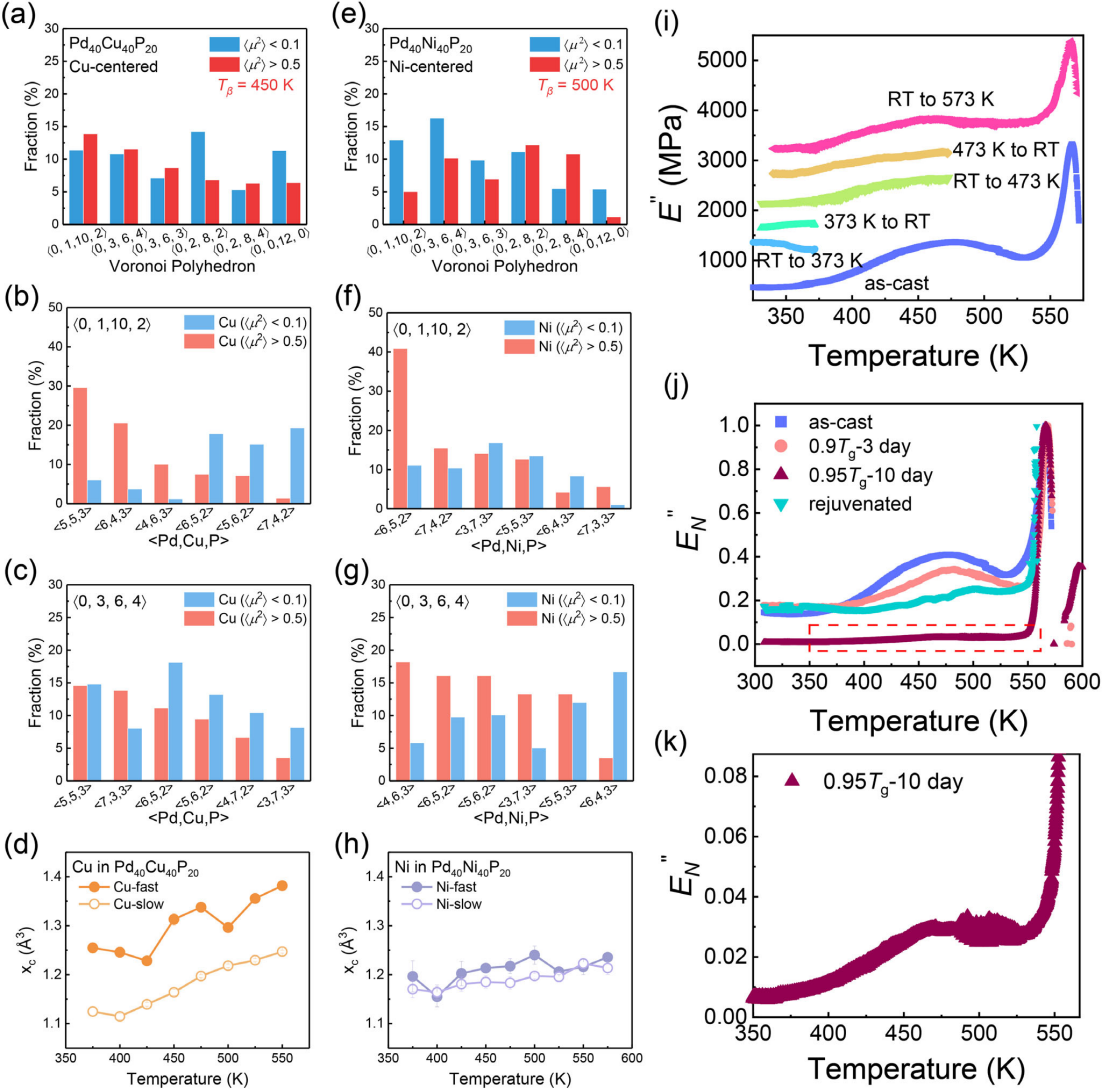
A comparison of the static structure of Pd_40_Cu_40_P_20_ and the Pd_40_Ni_40_P_20_ MGs, along with the cyclic‐heating evolution of DMA profiles for Pd_40_Cu_40_P_20_ MG. a) Fractions of major VP centered by Cu atoms at 450 K. Partial CN of Cu atoms in b) 〈0, 1,10, 2〉 and c) 〈0, 3, 6, 4〉 polyhedra in Pd_40_Cu_40_P_20_ MG. d) Variations in the *x*_c_ of Cu atoms with temperature rising. e) Fractions of major VP centered by Ni atoms at 500 K. Partial CN of Ni atoms in f) 〈0, 1,10, 2〉 and g) 〈0, 3, 6, 4〉 polyhedra in Pd_40_Ni_40_P_20_ MG. h) Variations in the *x*_c_ of Ni atoms with increasing temperature. i) Temperature dependences of loss modulus during cyclic heating first to 373 K and then to 473 K at 3 K min^−1^, 1 Hz, RT: room temperature. j) Normalized loss modulus for as‐cast, 0.9*T*_g_‐3 day annealed, 0.95*T*_g_‐10 day annealed, and rejuvenated samples. k) Enlarged view of the loss modulus curve for the 0.95*T*_g_‐10 day annealed sample below *T*_g_.

At the *T_β_* temperature, the fast Ni atoms in La_50_Al_15_Ni_35_ MG exhibit significantly higher fraction proportions <La, Al, Ni> of <8, 1, 0> and <7, 2, 0> within the 〈0, 3, 6, 0〉 polyhedron, and <8, 0, 2> and <8, 1, 1> in the 〈0, 3, 6, 1〉 polyhedron, compared to slow Ni atoms, indicating that more large‐sized La atoms in the first shell favor to form more free volume, thereby enhancing Ni mobility. In contrast, a greater number of La atoms does not accelerate Cu mobility in La_50_Al_15_Cu_35_ MG, likely due to the presence of additional Cu atoms in the local environment. B atoms surrounded by Zr atoms show decreased mobility, likely attributable to the strong Zr–B bonding interactions (*ΔH*_mix_ = −71 kJ mol^−1^). Fast P atoms in Pd_40_Cu_40_P_20_ MG exhibit a higher fraction of <4, 5, 0> and <3, 6, 0> within the 〈0, 3, 6, 0〉 polyhedron, and <5, 3, 0> within the 〈0, 4, 4, 0〉 polyhedron, compared to slow P atoms. This indicates that more Cu neighbors and fewer Pd neighbors enhance the mobility of P atoms, as Cu─P bonds are weaker (*ΔH*_mix_ = −17.5 kJ mol^−1^) than Pd─P bonds (*ΔH*_mix_ = −36.5 kJ mol^−1^). In Pd_40_Ni_40_P_20_ MG, both Pd‐P and Ni─P bonds (*ΔH*_mix_ = −34.5 kJ mol^−1^) restrict P atom mobility. MSD analysis further confirms that Cu diffuses faster than Ni, consistent with the weaker bonding constraints between P and Cu compared to those between P and Ni (Figure S10f–j, Supporting Information). **Figure** **5**a shows similar VP distributions around Cu atoms in both fast and slow states, except for icosahedral‐like structures such as 〈0, 2, 8, 2〉 and 〈0, 0, 12, 0〉, which occur more frequently around slow Cu atoms due to their dynamic hindrance. By contrast, Ni atoms exhibit more pronounced differences in VP distributions between fast and slow states (Figure 5e), suggesting that Cu atoms transition between states more readily, whereas Ni migration requires substantial structural rearrangements. Regarding local chemical environments, Cu atoms surrounded by more Pd and P atoms and fewer Cu atoms in their first atomic shell tend to exhibit higher mobility. For instance, fast Cu atoms show higher proportions of <5, 5, 3> and <6, 4, 3> chemical compositions within 〈0, 1, 10, 2〉 polyhedra and <7, 3, 3> within 〈0, 3, 6, 4〉 polyhedra compared to slow Cu atoms (Figure 5b,c). Conversely, fast Ni atoms more commonly have increased numbers of Ni neighbors, as observed for compositions such as <6, 5, 2> within 〈0, 1,10, 2〉, and <4, 6, 3> and <3, 7, 3> within 〈0, 3, 6, 4〉 polyhedra (Figure 5f,g). Similar trends appear in other VP types (Figure S25, Supporting Information). These analyses clearly demonstrate that local chemical compositions play a critical role in governing atomic dynamics.

### 3.2 Local Structure Contribution to the β‐Relaxation: Free Volume, CN, and Bond Length

Both elongated Cu─Pd bonds identified by XAFS analysis and the TTP‐like polyhedral configuration of P atoms surrounding Cu centers create additional local free volume and facilitate Cu mobility. In contrast, Ni atoms form denser local configurations due to higher Ni and P concentrations in their first shells. Stronger Ni─P bonding further restricts atomic mobility, resulting in a less pronounced *β*‐relaxation peak in Pd_40_Ni_40_P_20_ MG. Free volume analysis corroborates these observations, showing consistently larger free volumes for fast Cu atoms compared to Ni atoms across temperatures. Notably, a distinct peak in Cu atom free volume emerges ≈475 K, coinciding with the *β*‐relaxation and diffusivity maxima (Figure 5d,h). Further quantitative comparisons in La‐based MGs reveal more significant structural changes (in free volume, CN, and bond length compared with slow atoms) for fast Ni atoms (6.74%, −0.91%, and 2.38%) relative to fast Cu atoms (1.53%, 1.03%, and 1.52%). In Pd‐based MGs, fast P atoms in Pd_40_Cu_40_P_20_ exhibit larger average free volume (1.319 Å^3^), lower CN (8.26), and longer bond length (2.469 Å) than those in Pd_40_Ni_40_P_20_ MG (1.317 Å^3^, 8.29, and 2.413 Å; see **Table** **1** and Tables S1 and S2, Supporting Information). Similarly, Cu atoms show substantially larger average free volumes in both slow and fast states (1.290 and 1.403 Å^3^, an 8.76% increase) compared to Ni atoms (1.264 and 1.313 Å^3^, a 3.88% increase; see Table 1). Cu atoms also exhibit smaller average CN values and longer bond lengths than Ni atoms (Tables S1 and S2, Supporting Information). In Fe_86_Zr_8_B_6_ MG, fast B atoms similarly display greater free volumes than average. Collectively, these findings strongly support the conclusion that pronounced atomic mobility and *β*‐relaxation are locally facilitated by structurally looser TTP‐like polyhedral environments, such as 〈0, 3, 6, 0〉.

**Table 1:** Free volumes of various types of Voronoi polyhedron for the five studied MGs at their respective *T_β_*, along with the free volume increment of fast atoms relative to slow atoms in each alloy.

Alloys	Center atom	Voronoi Polyhedron	Slow atoms 〈*µ* ^2^〉 < 0.1 Å^2^	Fast atoms 〈*µ* ^2^〉> 0.5 Å^2^	Increment	*β *−peak
			Free Volume [Å^3^]		Δ%	
La_50_Al_15_Ni_35_	Ni	〈0, 3, 6, 0〉	1.543	1.596	3.43	pronounced
		〈0, 3, 6, 1〉	1.436	1.544	7.52	
		total	1.483	1.583	6.74	
La_50_Al_15_Cu_35_	Cu	〈0, 3, 6, 0〉	1.962	1.965	0.15	weak
		〈0, 2, 8, 1〉	1.576	1.600	1.52	
		total	1.630	1.655		1.53
Pd_40_Cu_40_P_20_	P	〈0, 3, 6, 0〉	1.105	1.118	1.18	pronounced
		〈0, 4, 4, 0〉	1.355	1.331	−1.77	
		total	1.219	1.319	8.20	
	Cu	〈0, 1,10, 2〉	1.260	1.444	14.60	
		〈0, 3, 6, 4〉	1.271	1.356	6.69	
		〈0, 2, 8, 4〉	1.167	1.248	6.94	
		〈0, 2, 8, 2〉	1.350	1.404	4.00	
		total	1.290	1.403	8.76	
Pd_40_Ni_40_P_20_	P	〈0, 3, 6, 0〉	1.090	1.137	4.31	weak
		〈0, 4, 4, 0〉	1.385	1.340	−3.25	
		total	1.308	1.317	0.69	
	Ni	〈0, 1,10, 2〉	1.368	1.372	0.29	
		〈0, 3, 6, 4〉	1.351	1.397	3.40	
		〈0, 2, 8, 4〉	1.247	1.310	5.05	
		〈0, 2, 8, 2〉	1.421	1.477	3.94	
		total	1.264	1.313	3.88	
Fe_86_Zr_8_B_6_	B	〈0, 3, 6, 0〉	0.466	0.471	1.07	pronounced
		〈0, 4, 4, 0〉	0.508	0.510	0.39	
		total	0.398	0.434	9.05	

### 3.3 Irreversible and Reversible β‐Relaxation

Additionally, we explore the *β*‐relaxation behaviors under thermal cycling using conventional DMA and DSC, with particular attention to the phenomenon of the shadow glass transition. First, an as‐cast Pd_40_Cu_40_P_20_ sample was continuously heated to 580 K at a rate of 3 K min^−1^ under a frequency of 1 Hz. A pronounced *β*‐relaxation peak appeared between ≈400 and 525 K, as indicated by the purple curve in Figure 5i. For comparison, another sample was heated under identical conditions to 373 K, a temperature below the onset of *β*‐relaxation, and subsequently cooled to room temperature inside the furnace. During the second heating cycle to 473 K (the peak temperature of *β*‐relaxation), the *β*‐relaxation hump was still detectable (green dots, Figure 5i). After another cooling cycle to room temperature, the third heating cycle was conducted to 573 K (pink dots, Figure 5i). Although the *β*‐relaxation peak persisted, its intensity slightly decreased compared to the as‐cast sample, indicating partial irreversibility upon cyclic heating.

Since rapid heating alone could not eliminate *β*‐relaxation entirely, we further investigated the effects of ex situ annealing treatments (Figure 5j). An as‐cast Pd_40_Cu_40_P_20_ sample was continuously heated beyond *T*_g_ at 3 K min^−1^ (purple curve). After annealing another sample at 0.9*T*_g_ (490 K) for 3 days, the *β*‐relaxation hump significantly weakened and shifted toward higher temperatures (coral curve). Based on prior studies linking *β*‐relaxation to free volume or open volume,^[^ ^7^, ^29^, ^30^, ^46^ ^]^ an additional as‐cast sample was annealed at 0.95*T*_g_ (520 K) for 10 days to ensure thorough free volume annihilation. Consequently, the *β*‐relaxation became very weak (deep red curve, Figure 5j), confirming the removal of its irreversible components. Adopting the method by Zhao et al.^[^ ^31^ ^]^ to verify the reversible component of *β*‐relaxation, we rapidly reheated the 0.95*T*_g_‐10 day annealed sample to near *T*_g_ (heating rate: 10 K min^−1^) and subsequently quenched it at 100 K min^−1^ to room temperature. The rejuvenated sample exhibited partial recovery of the *β*‐relaxation peak (cyan inverted triangles, Figure 5j). As illustrated in Figure 5k, although the *β*‐relaxation peak of the 0.95 *T*_g_‐10 day annealed sample was significantly reduced, its peak position (≈480 K) remained distinct and did not merge with the *α*‐relaxation even upon magnifying the temperature range below 550 K. Previous studies have identified the shadow glass transition observed through DSC as a thermodynamic signature correlated with *β*‐relaxation.^[^ ^17^, ^31^ ^]^ The DSC observations in Figure S26 (Supporting Information) clearly demonstrate that heating beyond the shadow glass transition progressively weakens its intensity and shifts its peak toward higher temperatures, consistent with the enthalpy relaxation process, attributed mainly to free volume annihilation.^[^ ^7^, ^29^, ^30^ ^]^

Zhao et al.^[^ ^31^ ^]^ previously suggested that extended annealing gradually shifts the shadow glass transition peak to higher temperatures until it merges with the main glass transition, forming an overshoot above *T*_g_. In our experiments, however, both a shadow glass transition below *T*_g_ and an overshoot above *T*_g_ coexist in the 0.9 *T*_g_‐3 day annealed sample, indicating that the overshoot primarily results from free volume annihilation (enthalpy relaxation) rather than from migration of the shadow glass transition peak. Furthermore, the rejuvenated sample exhibited a reappearance of the shadow glass transition peak below *T*_g_, confirming partial reactivation of *β*‐relaxation. Collectively, our structural, modulus, and thermal analyses clearly demonstrate that local atomic structural rearrangements strongly influence *β*‐relaxation behaviors in MGs.

Over the past 25 years, substantial progress has been made in understanding *β*‐relaxation in metallic glasses (MGs), as summarized in Figure S27 (Supporting Information).^[^ ^2^, ^3^, ^4^, ^5^, ^6^, ^7^, ^8^, ^11^, ^17^, ^18^, ^21^, ^22^, ^28^, ^29^, ^30^, ^31^, ^46^, ^47^, ^48^, ^49^, ^50^, ^51^, ^52^, ^53^, ^54^, ^55^, ^56^, ^57^, ^58^, ^59^, ^60^, ^61^, ^62^, ^63^, ^64^, ^65^, ^66^, ^67^, ^68^, ^69^, ^70^, ^71^, ^72^, ^73^, ^74^, ^75^, ^76^, ^77^, ^78^, ^79^, ^80^, ^81^, ^82^, ^83^, ^84^ ^]^ The research trajectory has advanced from early characterizations using DMA and the enthalpy‐relaxation signals captured by DSC, through the identification of the so‐called “shadow glass transition” also manifested in DSC, to more recent insights provided by molecular dynamics simulations. In this study, we introduce a straightforward structural characterization method that enables deeper probing of *β*‐relaxation, thereby providing a new avenue to address long‐standing questions in this field. Importantly, the proposed method is applicable not only to systems with pronounced *β*‐relaxation but also to more general systems where *β*‐relaxation is weak or barely discernible. By combining diffusion behavior, DMA responses, synchrotron structural signatures, and molecular dynamics simulations, we establish a consistent framework that demonstrates both the robustness and the broad applicability of the methodology, while underscoring its general significance and novelty.

In addition, to broaden the implications, controlling *β*‐relaxation dynamics offers a promising pathway for optimizing the mechanical properties of metallic glasses—particularly ductility and fracture toughness. In La‐based MGs, pronounced *β*‐relaxation promotes homogeneous STZ activation, delaying shear band formation and enhancing plasticity.^[^ ^2^ ^]^ To suppress shear banding, increasing TTP polyhedra via microalloying (e.g., with B or P) can homogenize shear and enhance structural uniformity. In addition to compositional design, the intensity of *β*‐relaxation can be modulated through thermal annealing/activation: sub‐*T*_g_ annealing reduces its strength and shifts the peak, while cold working or thermal rejuvenation enhances it via free volume increase. These approaches offer complementary routes to tailor mechanical properties of MGs.

## 4 Conclusion

In summary, we utilize in situ high‐temperature X‐ray diffraction and absorption techniques to experimentally characterize *β*‐relaxation in terms of structural signals and to correlate these with atomic packing rearrangements. Taking Pd_40_Cu_40_P_20_ MG as a model system, we find that *β*‐relaxation is strongly influenced by the evolution of the Cu/Pd‐centered atomic packing. MD simulations reveal that *β*‐relaxation is closely related to fast atomic motions, which are directly accelerated by increasing local atomic free volume with reduced coordination numbers and extended average bond lengths. Systems exhibiting strong *β*‐relaxation are not only related to their polyhedral types but also elemental constitutions. Furthermore, cyclic heating experiments highlight both the irreversible and reversible features of *β*‐relaxation, as evidenced by changes in loss modulus and thermodynamic signals. Our findings provide a robust experimental and theoretical basis for systematically exploring *β*‐relaxation via standard structural characterization methods, enabling deeper insights into the structural origins and universal characteristics. Ultimately, these results enhance our understanding of the reversible and irreversible nature of *β*‐relaxation, which are crucial for tailoring the stability and mechanical properties of MGs.

## 5 Experimental Section

### Fabrication of Materials

First, high‐purity red phosphorus and palladium were heated in a vacuum quartz tube to prepare a pre‐alloy with a nominal composition of Pd_65_P_35_; the experimental protocol details can be referred to in the literature.^[^ ^25^ ^]^ To eliminate impurities and oxidation, the B_2_O_3_ flux treatment for pre‐alloys was inductively heated under vacuum more than ten times.^[^ ^85^ ^]^ Subsequently, high‐purity Pd/Cu (Ni) monomers were added to the pre‐alloy according to the target atomic ratios. The final ingots were obtained by arc melting, with at least four remelting cycles to improve chemical homogeneity, followed by an additional B_2_O_3_ flux treatment to further purify the Pd_40_Cu_40_P_20_ and Pd_40_Ni_40_P_20_ ingots. The ingots for other MGs of La_50_Al_15_ (Ni/Cu)_35_, Fe_86_Zr_8_B_6_, CuZr_2_, Cu_50_Zr_50_, Cu_64_Zr_36_, and Cu_46_Zr_46_Al_8_ were prepared by arc melting under high vacuum. Subsequently, all melts were rapidly quenched using melt spinning onto a rotating copper wheel at a surface speed of 30 m s^−1^ in an argon atmosphere, yielding ribbon samples with a thickness of ≈40 µm and a width of 3 mm.

### High‐Energy X‐ray Diffraction

The ribbon samples were cut into pieces and loaded into capillaries with an inner diameter of 1.5 mm (wall thickness ≈0.01 mm) and then vacuum‐sealed at ≈2.0×10^−5^ mbar. High‐energy X‐ray diffraction experiments were performed at the BL12SW of Shanghai Synchrotron Radiation Facility (SSRF), China, with the wavelength of ≈0.1390 Å and the beam size of ≈0.3×0.3 mm^2^. The samples were heated continuously at a rate of 10 K min^−1^ to below *T*_g_, and diffraction data were recorded with a Pilatus 2M detector. The exposure time was 1 s, and 20 diffraction patterns were summed for each data set. The scattering intensities *I*(*q*) were obtained after background subtraction and integration^[^ ^86^ ^]^ using the Fit2D software. Standard procedures were applied to extract the static structure factor *S*(*q*) and the pair distribution function *G*(*r*), including appropriate background subtraction and corrections using the GSAS‐II software package.^[^ ^87^ ^]^

In X‐ray scattering experiments, the structure factor is defined as:

(1) $$S\left(q\right)=\frac{I\left(q\right)-\langle f^{2}\left(q\right)\rangle -K}{{\langle f(q)\rangle }^{2}}$$

Here, *q* is the scattering vector, defined as *q* = 4πsin θ/λ, *I*(*q*) is the diffraction intensity obtained from the integration of the experimental spectrum, *f*(*q*) is the atomic scattering factor, and *K* represents the Compton (inelastic) scattering correction factor. By performing a Fourier transform of the structure factor *S*(*q*), the pair distribution function *G*(*r*) can be obtained, which is defined as:

(2) $$G\left(r\right)=4\pi r[\rho \left(r\right)-\rho_{0}]$$

Here, ρ(*r*) represents the atomic density within a spherical shell at a distance *r* from the central atom.

The room‐temperature data for Pd_40_(Cu/Ni)_40_P_20_, La_50_Al_15_(Ni/Cu)_35_, Fe_86_Zr_8_B_6_, CuZr_2_, Cu_50_Zr_50_, Cu_64_Zr_36_ and Cu_46_Zr_46_Al_8_MGs were obtained at beamline 11‐ID‐C of the Advanced Photon Source (APS), Argonne National Laboratory (Chicago, USA), using an incident X‐ray energy of 105.7 keV and a beam size of ≈0.5 × 0.5 mm^2^.

### X‐ray Absorption Fine Structure Spectrum

The extended X‐ray absorption fine structure (EXAFS) experiments on the Pd K‐edge (24350 eV) and Cu K‐edge (8979 eV) were conducted at TPS 44A of the National Synchrotron Radiation Research Center (NSRRC), Taiwan, with a beam size of ≈0.1×0.3 mm^2^. The reference standard Cu and Pd foils were measured simultaneously to calibrate the incident photon energy. The samples in capillaries were heated continuously at a rate of 10 K min^−1^ to below *T*_g_. Both the heating source and thermocouple were located 1 mm away from the sample to ensure reliable temperature measurement. The exposure time was 0.5 s, and 20 patterns were summed for each data set. The EXAFS data were Fourier transformed from *k*‐space spectra to *R*‐space curves after background subtraction and energy calibration, using the Athena software package.^[^ ^88^ ^]^

### DMA and DSC Analyses

A TA‐Q800 DMA was used to probe *β*‐relaxation by film stretching mode. Measurements were performed at a heating rate of 3 K min^−1^ and a frequency of 1 Hz. DSC measurements were carried out using a Netzsch 404C and a TA DSC 2500 instrument at a heating rate of 10 K min^−1^ under a flowing argon atmosphere of 25 mL min^−1^. The mass of samples was kept at ≈20 mg to ensure the accuracy of the data.

### Molecular Dynamic Simulations

First‐principles calculations based on ab initio molecular dynamics (AIMD) simulations were conducted using the Vienna Ab‐initio Simulation Package (VASP).^[^ ^89^ ^]^ The configuration of Pd‐Cu‐P at the initial 300 K^[^ ^25^ ^]^ was progressively heated to 550 K in 10 K steps, and at each temperature, the pressure was controlled to be almost zero as well as the energy constant by adjusting the volume of the simulation box, and the system was equilibrated for 10 000 steps at each temperature to generate sufficient configurations for analysis.

For the Pd_40_Ni_40_P_20_, La_50_Al_15_(Ni/Cu)_35_, Fe_86_Zr_8_B_6_ MGs, the simulations were carried out in cubic boxes with periodic boundary conditions containing 200 atoms distributed according to their nominal compositions. These systems were first heated to 2000 K and relaxed for 10 000 steps with a time step of 3 fs to eliminate any memory effects. Subsequently, the temperature was gradually reduced to 1500, 1000, 700, 500, and 300 K at a cooling rate of 0.1 K step^−1^. At each temperature, the pressure was held near zero, and the energy constant was as above. The same heating rate was used to increase the temperature up to the respective *T*_g_ based on experimental data. The systems were relaxed for 10 000 steps at every 25 K increment, and the configurations of the final 8000 steps were used for quantitative analysis.

For the Cu_50_Zr_50_, Cu_64_Zr_36_, CuZr_2_, Cu_46_Zr_46_Al_8_ MGs, MD simulations were performed using the LAMMPS software package developed by Sandia National Laboratory in conjunction with the embedded atom potential developed by Mendelev et al. The simulation system employs 3D periodic boundary conditions (PBCs) with a cubic box of 5.5 × 5.5 × 5.5 nm^3^ (x‐y‐z) containing 9472 atoms for Cu‐Zr systems and 10001 atoms for Cu‐Zr‐Al systems. The MD simulations were run in the NPT ensemble (constant number of atoms, pressure, and temperature) with a time step of 0.001 ps. For the pure MD scheme, the system was first relaxed at 2000 K (much above the melting point of the alloy) for 6 ns to eliminate the periodicity and thermal history of crystals, then was quenched to 300 K at a cooling rate of 10^11^ K s^−1^ to obtain a fast‐quenched sample. The same heating rate was used to increase the temperature up to the respective *T*_g_ based on experimental data. The systems were relaxed for 1 000 000 steps at every 20 K increment to obtain sufficient configurations for analysis.

Free volume was calculated using a method analogous to finite element analysis. The 3D periodic simulation box was divided into equal‐sized cubes based on a defined unit volume. The radius of each atom was adjusted by multiplying its actual radius by a magnification factor of 1.1 to minimize the contribution of very small free volumes to atomic motion. The cutoff radii were set at 4.5 Å for Ni/Cu in La_50_Al_15_(Ni/Cu)_35_ MGs, 3.6 Å for Cu/Ni and 3.0 Å for P in Pd_40_(Cu/Ni)_40_P_20_ MGs, 2.9 Å for B in Fe_86_Zr_8_B_6_ MG. 3.81 Å for Cu in Cu_50_Zr_50_ MG, 3.75 Å for Cu in Cu_64_Zr_36_ MG, 3.90 Å for Cu in CuZr_2_ MG, 3.63 Å for Cu in Cu_46_Zr_46_Al_8_ MG. The number of unoccupied cubes within these cutoff spheres was assessed, and the total volume, along with the number of atoms, was calculated. The free volume for a given central atom was obtained by dividing the total volume by the number of atoms within the cutoff. The precision of the final free volume calculation was influenced by the unit volume, which in this work was set to 0.1 Å per side.

The MSD is defined as follows:

(3) $$\langle r^{2}\left(t\right)\rangle =\frac{1}{N}\langle \sum_{i=1}^{N}{\left|r_{i}\left(t\right)-r_{i}\left(0\right)\right|}^{2}\rangle$$

where *N* is the total atom number in a system, *r_i_* (0) is the initial position of the atom *i*. *r_i_* (*t*) is the position of the atom *i* at time *t*. The self‐diffusion coefficient(*D*) can be calculated by the MSD based on the Einstein equation,

(4) $$D=\lim_{t\to \infty }\frac{1}{6t}\langle r^{2}\left(t\right)\rangle$$

The Gumbel function used for fitting the free volume distribution is as follows:

(5) $$y=y_{0}+Ae^{\left(-e^{\left(-z\right)}-z+1\right)},\;z=\left(x-x_{c}\right)/w$$

where *y*_0_ is the value of *y* when *A* = 0. *A* is the amplitude coefficient. *x*_c_ is the location parameter, which determines the center of the function. *w* is the scale parameter, which controls the width or shape of the function.

### Statistical Analysis

As mentioned above, all experimental data were analyzed using standard statistical and numerical methods. The diffraction and EXAFS data were processed through background subtraction, normalization, and Fourier transformation using Fit2D, GSAS‐II, and Athena software. For DSC and DMA measurements, the heating rate, gas atmosphere, and sample mass—were strictly controlled to ensure reproducibility and data consistency. In the MD and AIMD simulations, all physical quantities, such as mean‐square displacement (MSD), diffusion coefficient (*D*), and free‐volume distribution, were obtained by averaging over multiple statistically independent configurations.

## Conflict of Interest

The authors declare no conflict of interest.

## Supporting information



Supporting Information

## Data Availability

The data that support the findings of this study are available from the corresponding author upon reasonable request.
