# Supplementary material for: Atomic‐Level Structural Characteristics of β‐Relaxation in Metallic Glasses
Source: Adv Sci (Weinh). 2025 Nov 9;13(5):e18424. doi: 10.1002/advs.202518424 (PMC12849962; doi:10.1002/advs.202518424)
Supplement: Supplementary file 1 — Supporting Information [file ADVS-13-e18424-s001.docx]

Supplementary Materials for

**Atomic-level Structural Characteristics of *β*-relaxation in Metallic Glasses**

Tianding Xu^1,2,#^, Jinquan Zhou^1,2,#^, Xiao-Dong Wang^1,2*^, Ke Yang^3^, Qing-Ping Cao^1,2^, Dong-Xian Zhang^1,2,4^, Chih-Wen Pao^5*^, Jian-Zhong Jiang^1,2,6*^

*^1^International Center for New-Structured Materials (ICNSM), Laboratory of New-Structured Materials, Zhejiang University, Hangzhou, 310027, China.*

*^2^State Key Laboratory of Silicon Materials, and School of Materials Science and Engineering, Zhejiang University, Hangzhou, 310027, China.*

*^3^Shanghai Synchrotron Radiation Facility, Zhangjiang Lab, Shanghai Advanced Research Institute, Chinese Academy of Science, Shanghai 201210, China.*

*^4^State Key Laboratory of Modern Optical Instrumentation, Zhejiang University, Hangzhou, 310027, China.*

*^5^National Synchrotron Radiation Research Center, Hsin-Ann Road, Hsinchu Science Park, Hsinchu, 30076, Taiwan.*

*^6^Key Laboratory of Silicon-based Materials, The Ministry of Education, Key Laboratory of Automotive Glass of Fujian, Smart Automotive Glass Engineering Research Center of Fujian, and School of Materials Science and Engineering, Fuyao University of Science and Technology, Fuzhou, 350109, China.*

# These authors contributed equally to this work.

* Corresponding authors.

*E-mail addresses:* Wang, X.D. ([wangxd@zju.edu.cn](mailto:wangxd@zju.edu.cn)), Pao, C.W. ([pao.cw@nsrrc.org.tw](mailto:pao.cw@nsrrc.org.tw)), Jiang, J.Z. ([jiangjz@fyust.edu.cn](mailto:jiangjz@fyust.edu.cn) and [jiangjz@zju.edu.cn](mailto:jiangjz@zju.edu.cn)).

**
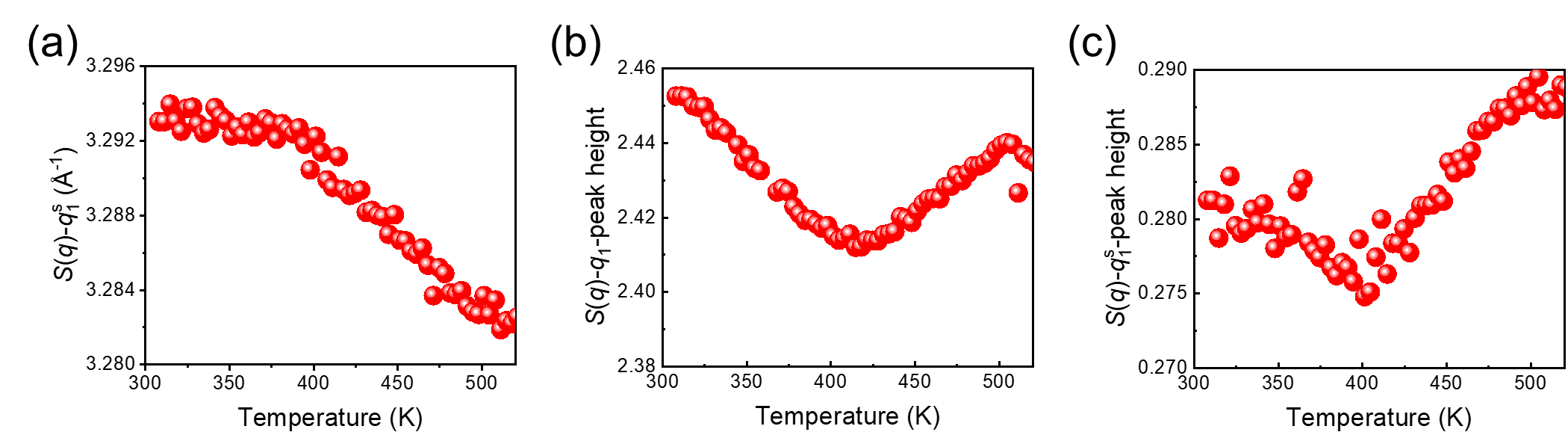
**

**Figure S1.** The temperature dependence of the first shoulder peak position and main and shoulder peak heights of *S*(*q*). (a) shoulder peak position *q*_1_^s^, (b) main peak height and (c) shoulder peak height of *S*(*q*).

**
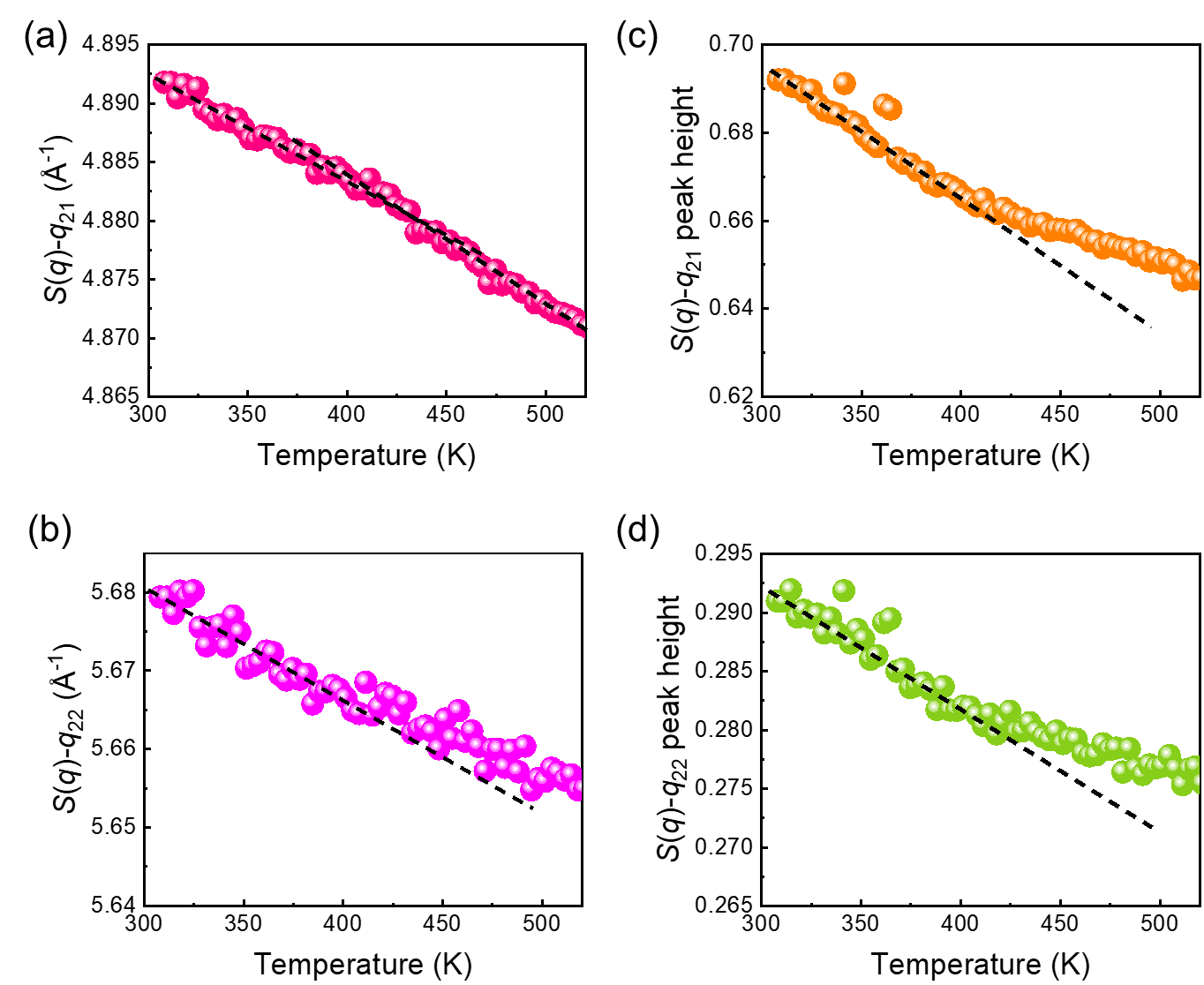
**

**Figure S2.** The temperature dependence of the second main and shoulder peak positions and heights of *S*(*q*). (a) main peak position *q*_21_, (b) shoulder peak position *q*_22_, (c) main peak height and (d) shoulder peak height of *S*(*q*).

**
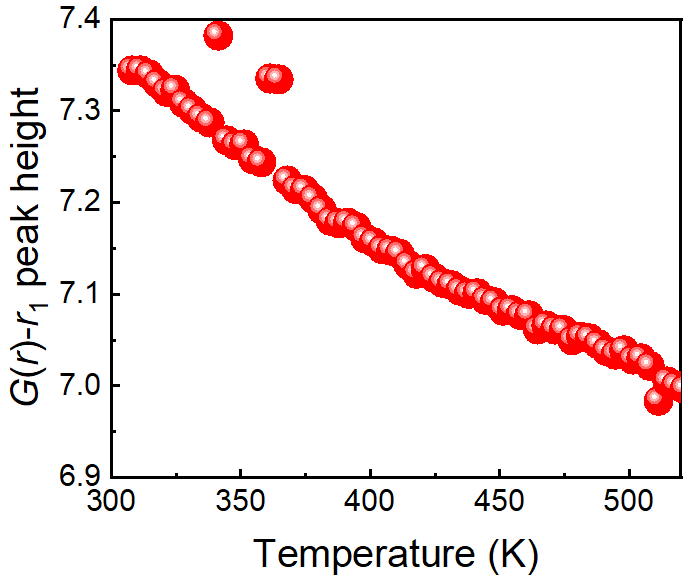
**

**Figure S3.** The temperature dependence of the first peak height of *G*(*r*).

**
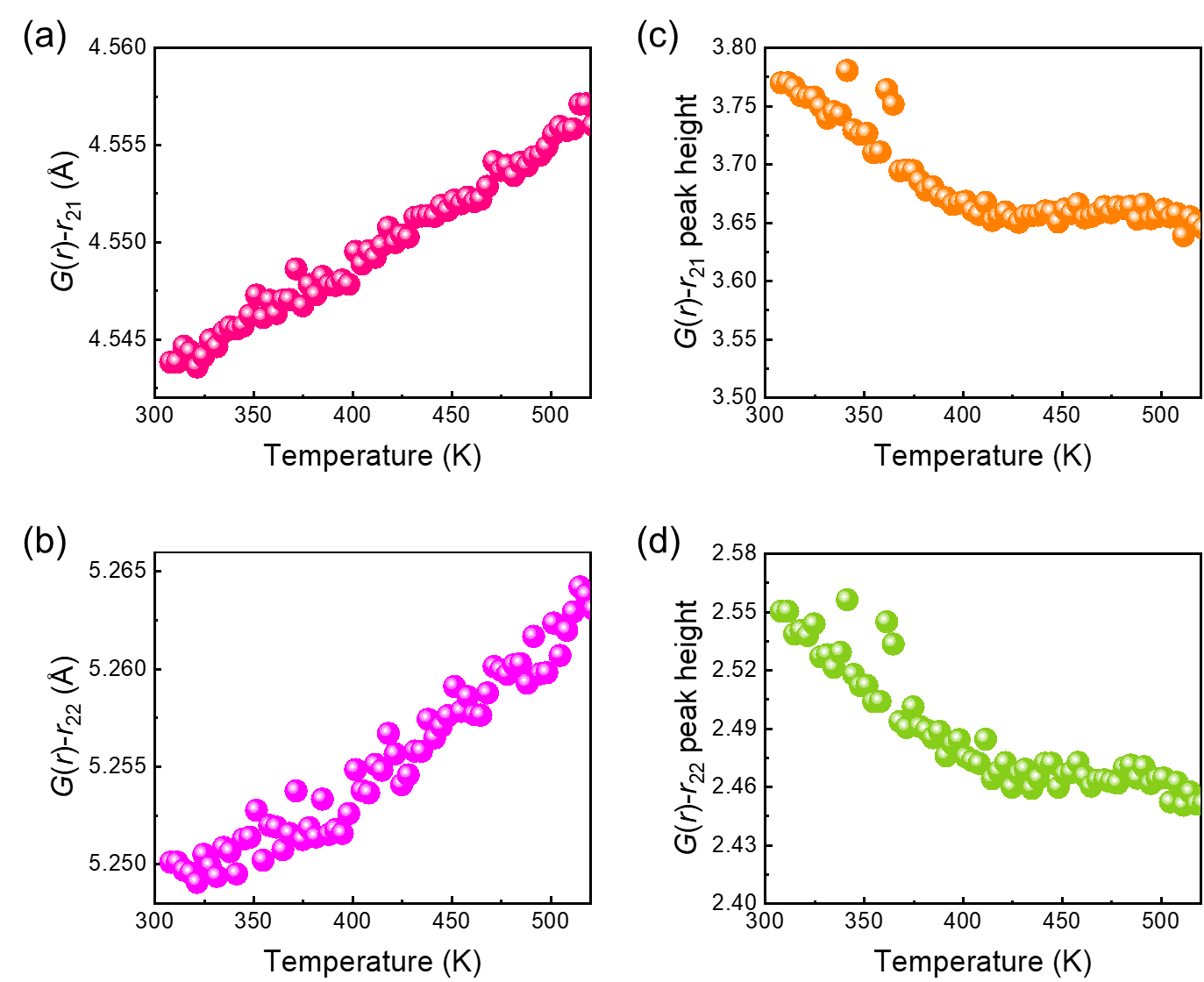
**

**Figure S4.** The temperature dependence of the second main and shoulder peak positions and heights of *G*(*r*). (a) main peak position *r*_21_, (b) shoulder peak position *r*_22_, (c) main peak height and (d) shoulder peak height of *G*(*r*).


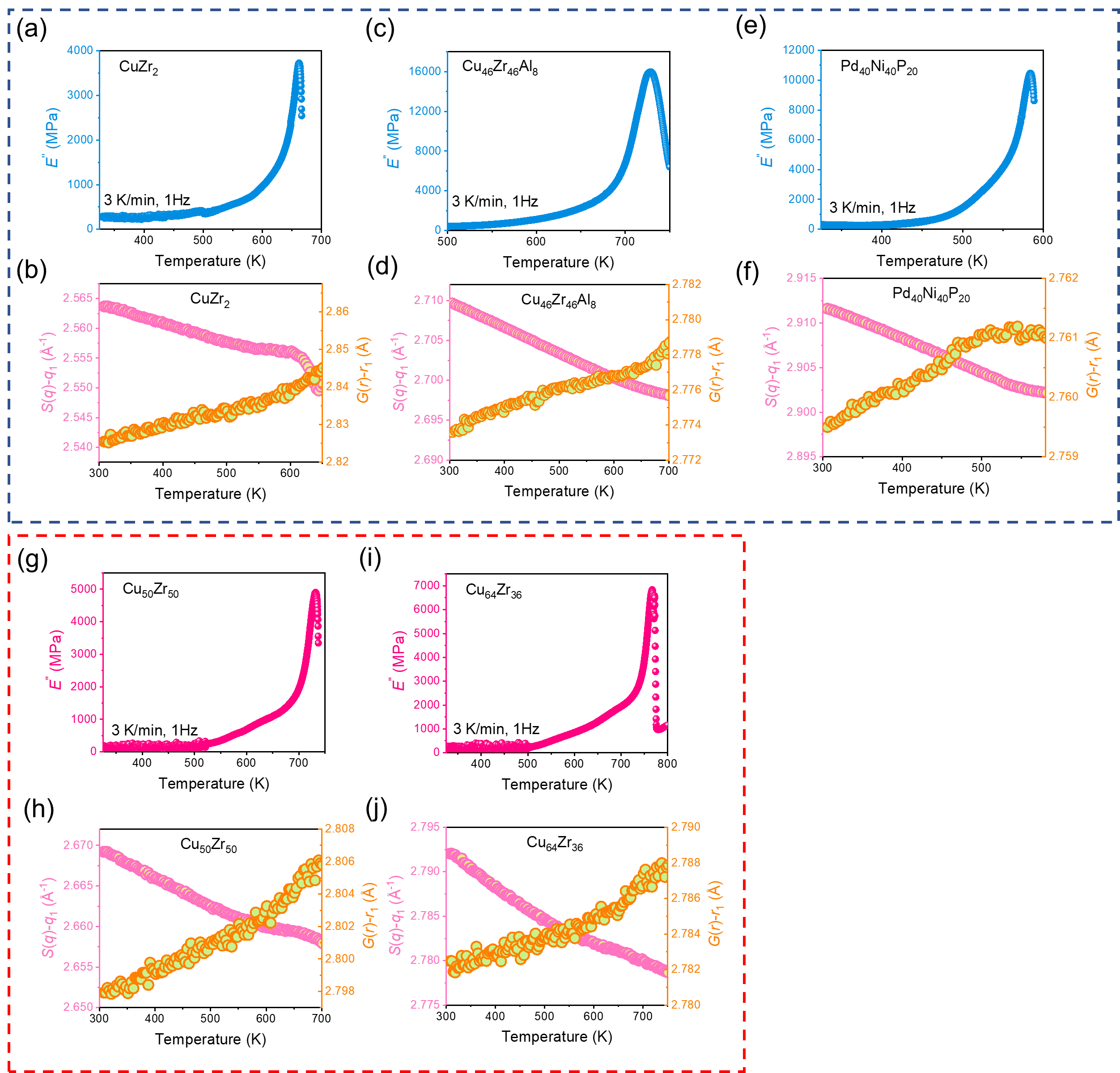


**Figure S5.** DMA profiles and the structural evolution associated with *β*-relaxation for (a,b) CuZr_2_, (c,d) Cu_46_Zr_46_Al_8_, (e,f) Pd_40_Ni_40_P_20_, (g,h) Cu_50_Zr_50_, and (i,j) Cu_64_Zr_36_ MGs. The regions within the blue dashed boxes correspond to systems with negligible *β*-relaxation, whereas those within the red dashed boxes correspond to systems in which *β*-relaxation is discernible.


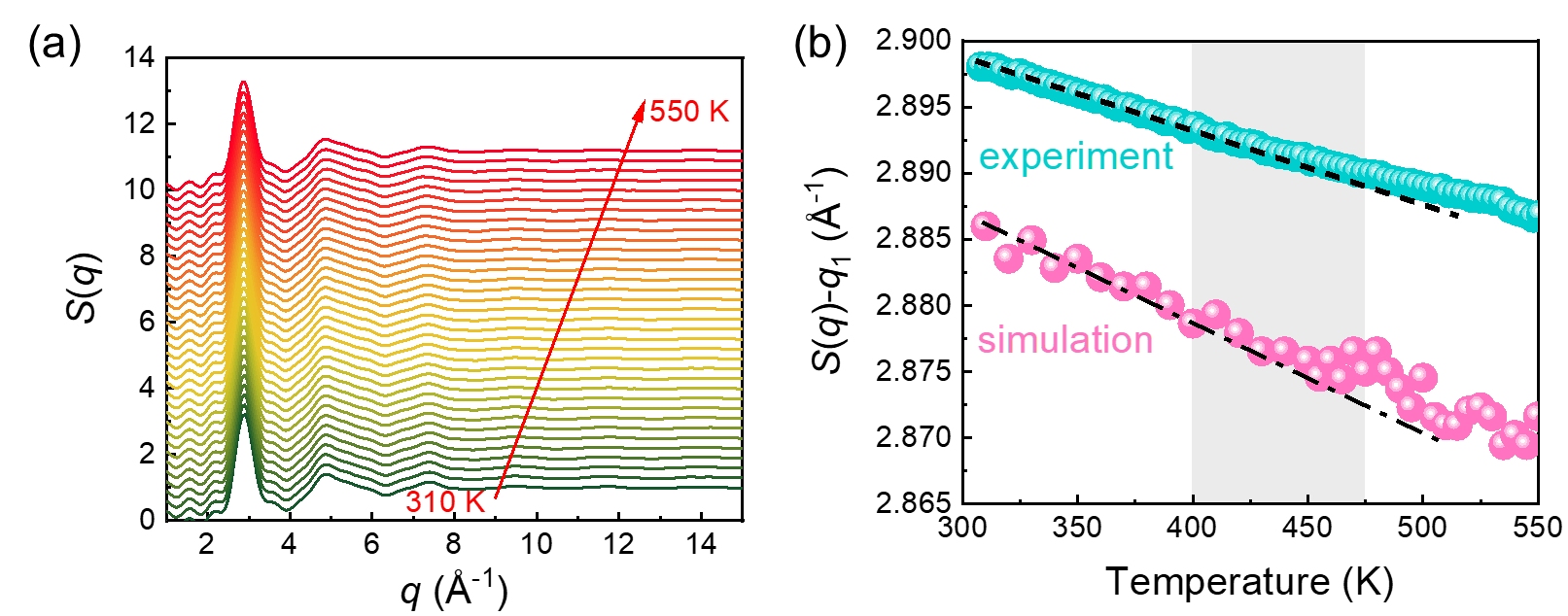


**Figure S6.** The temperature-dependent structure factor from simulation. (a) Simulated structure factor *S*(*q*) in the temperature range of 310-550 K. (b) Comparisons between the simulation (pink balls) and the experimental (cyan balls) data for *S*(*q*).


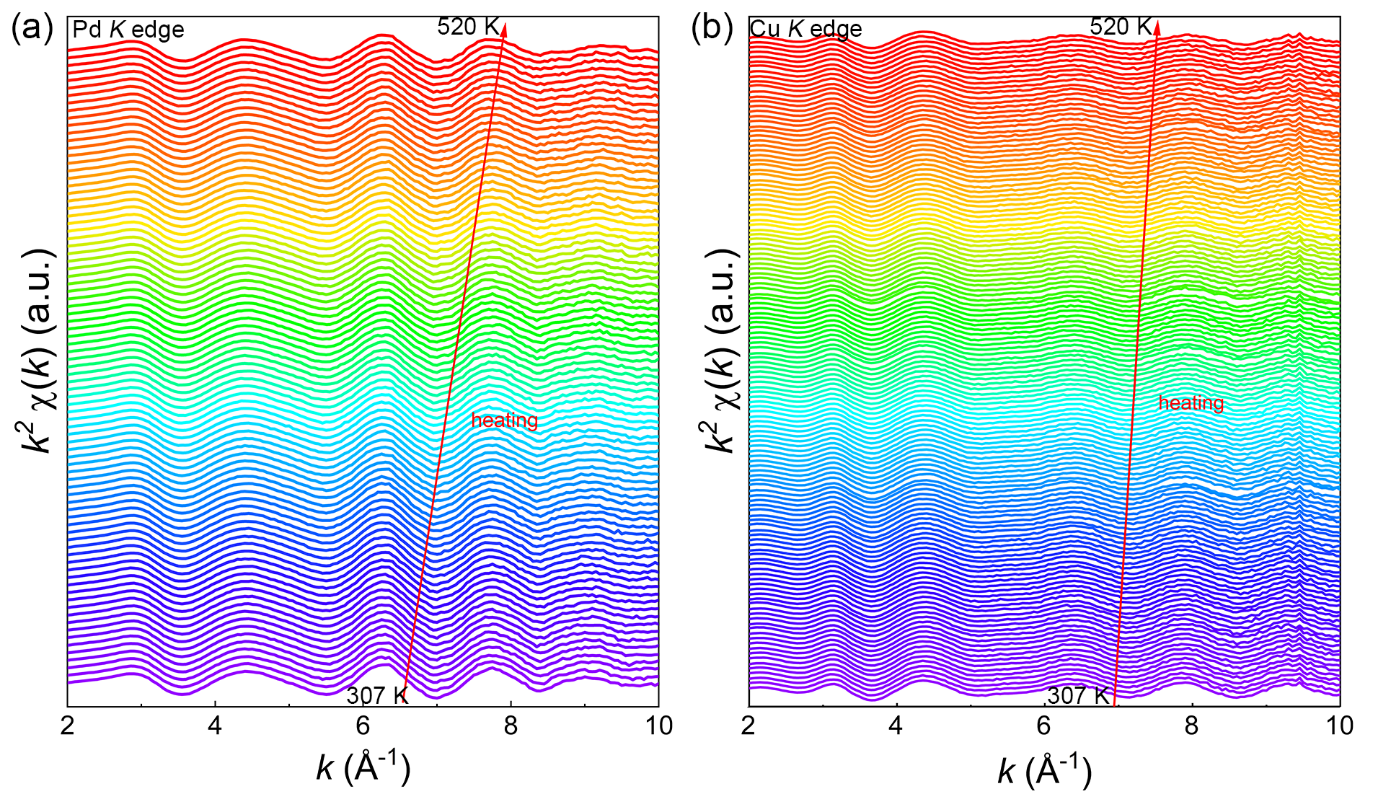


**Figure S7.** The peak evolution in situ high-temperature XAFS experiments at Cu/Pd *K* edges. (a) Temperature-dependent Cu *K*-edge X-ray absorption fine structure *k*^2^-weighted spectra measured in the range of 307-520 K. (b) Temperature-dependent Pd *K*-edge X-ray absorption fine structure *k*^2^-weighted spectra measured in the range of 307-520 K.


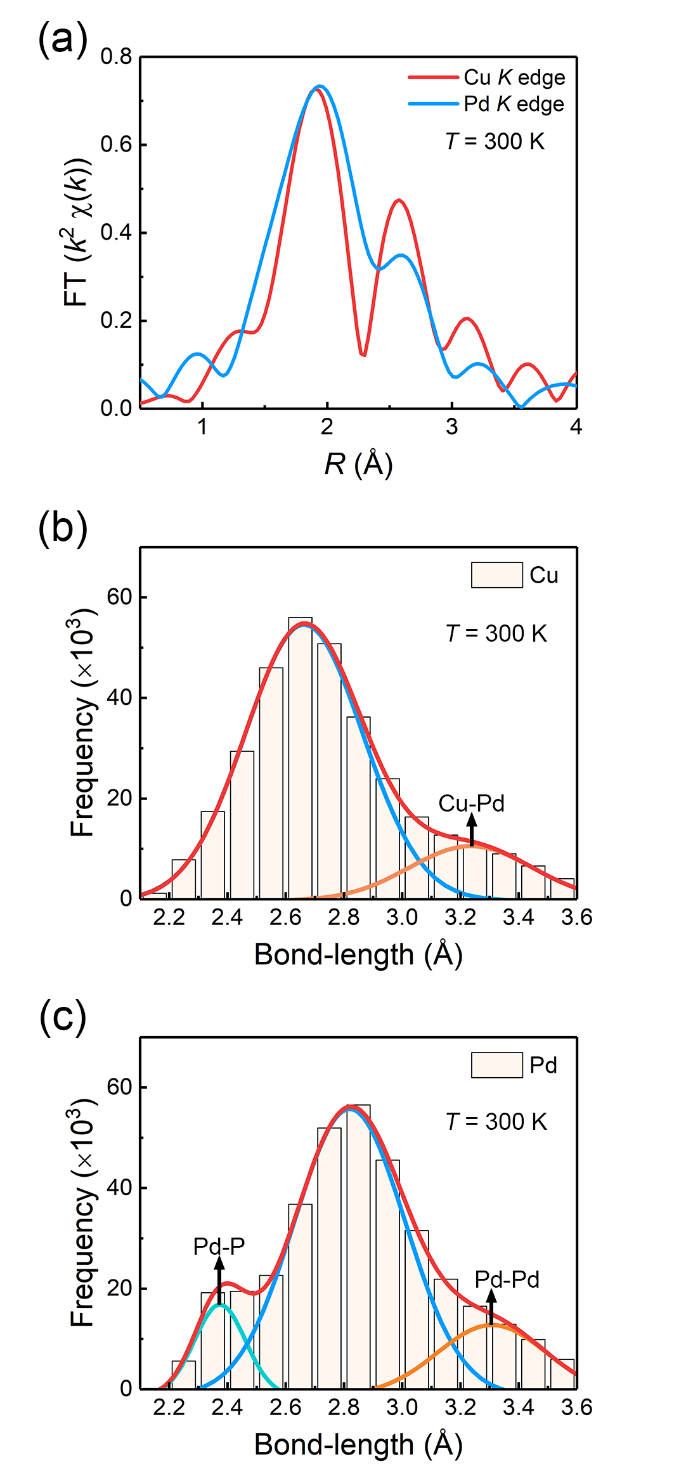


**Figure S8.** Local atomic structures of the studied Pd_40_Cu_40_P_20_ MGs. (a) *k*^2^-weighted *K*-edge Fourier transform profiles of Cu and Pd *K* edge at 300 K from EXAFS experiments. Gauss fitting analysis of bond-length distribution for (b) Cu and (c) Pd centered atoms obtained from AIMD simulation at 300 K.

**
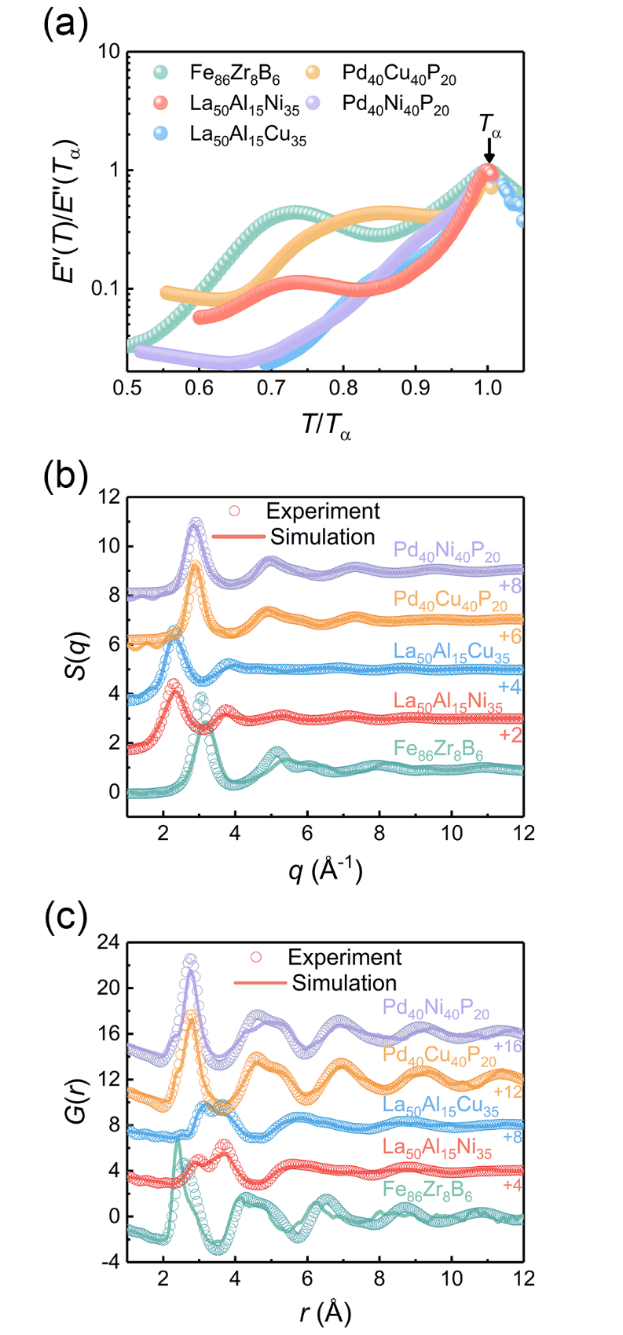
**

**Figure S9.** Dynamic mechanical analysis and structural comparison of XRD and AIMD simulations for the five MGs at 300 K. (a) The relationship between $E^{"}/E_{0}^{"}$ and $T/T_{\alpha}$ at a heating rate of 3 K/min and a frequency of 1 Hz. Comparisons of (b) structure factor *S*(*q*) and (c) pair distribution functions *G*(*r*) between high-energy X-ray diffraction (XRD; circles) and Ab initio Molecular Dynamics (AIMD; solid curves) simulation data for La_50_Al_15_Ni_35_, Pd_40_Cu_40_P_20_, La_50_Al_15_Cu_35_, Pd_40_Ni_40_P_20_ and Fe_86_Zr_8_B_6_ metallic glasses (MGs).


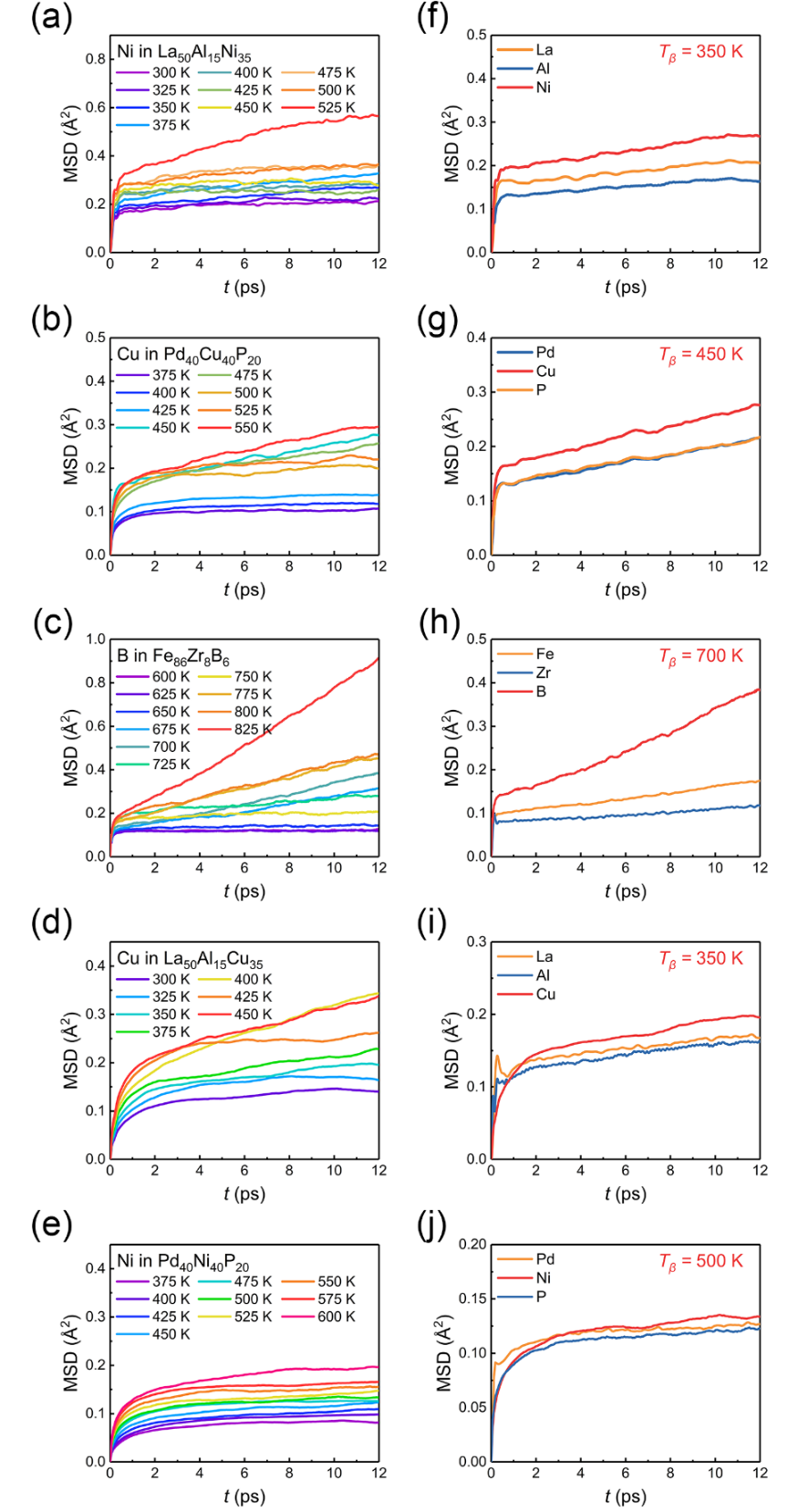


**Figure S10.** The partial mean-squared displacement (MSD) for selected atoms. (a) Ni in La_50_Al_15_Ni_35_, (b) Cu in Pd_40_Cu_40_P_20_, (c) B in Fe_86_Zr_8_B_6_, (d) Cu in La_50_Al_15_Cu_35_, and (e) Ni in Pd_40_Ni_40_P_20_. Partial MSD for (f) La, Al, Ni, (g) Pd, Cu, P, (h) Fe, Zr, B, (i) La, Al, Cu, and (j) Pd, Ni, P atoms in studied alloys at their own *T_β_*.
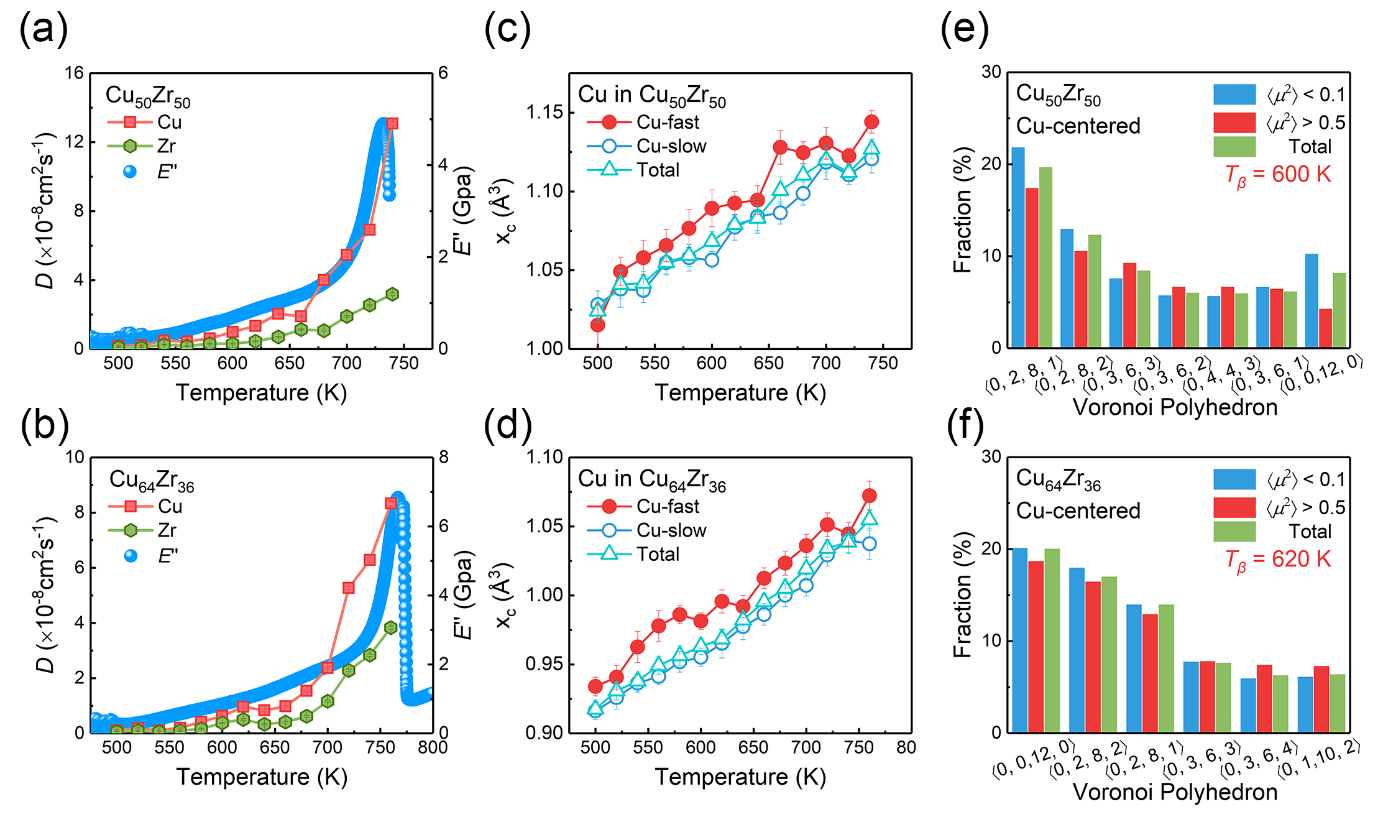


**Figure S11.** (a)-(b) Relationship between the self-diffusion coefficient (*D*) and loss modulus (*E*’’). (c)-(d) The fitted peak positions (*x*_c_) of the free volume distribution. (e)-(f) Fractions of major Voronoi polyhedra centered by smallest atoms (fast (〈*μ*^2^〉 > 0.5 Å^2^) and slow (〈*μ*^2^〉 < 0.1 Å^2^)) at *T_β_* of their own in Cu_50_Zr_50_ and Cu_64_Zr_36_ MGs.


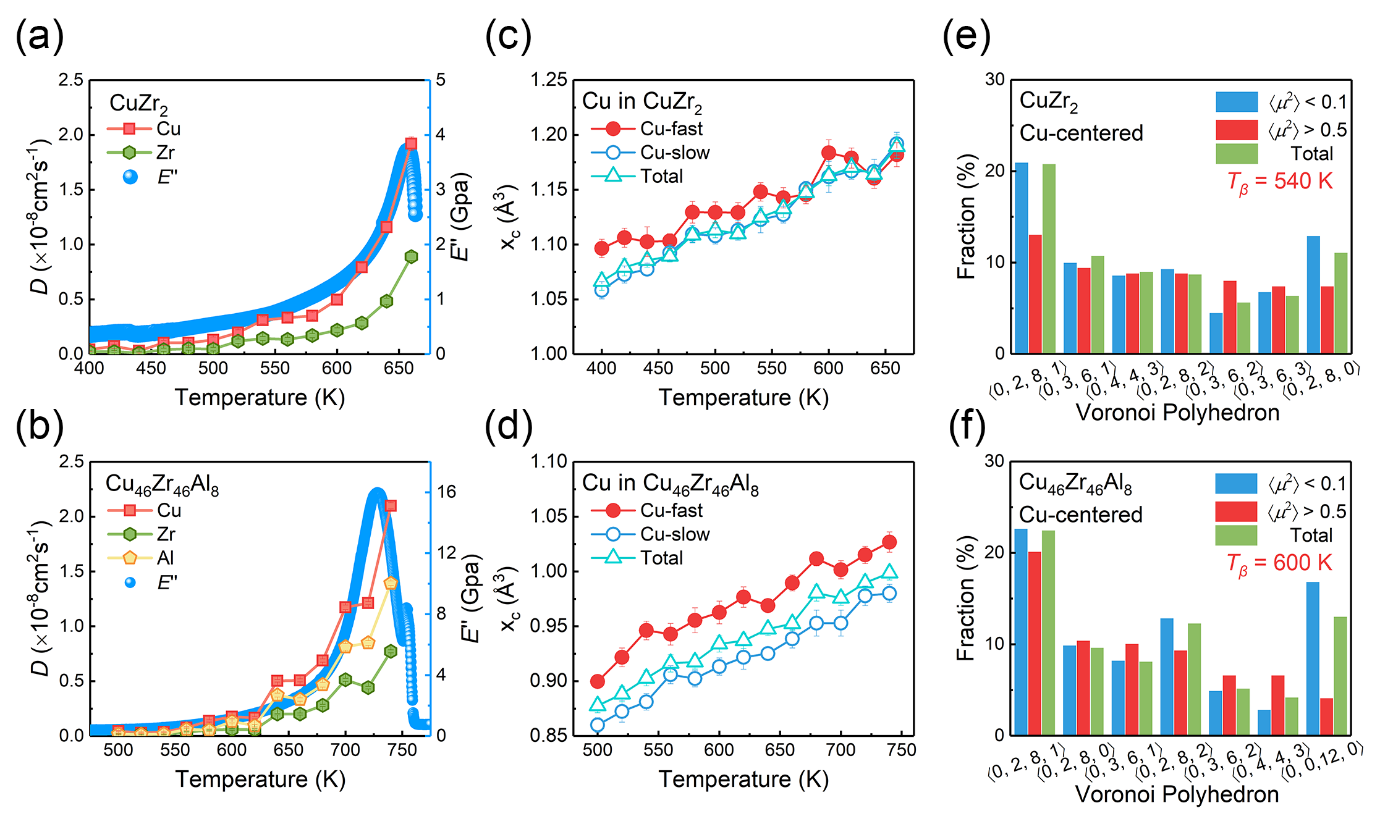


**Figure S12.** (a)-(b) Relationship between the self-diffusion coefficient (*D*) and loss modulus (*E*’’). (c)-(d) The fitted peak positions (*x*_c_) of the free volume distribution. (e)-(f) Fractions of major Voronoi polyhedra centered by smallest atoms (fast (〈*μ*^2^〉 > 0.5 Å^2^) and slow (〈*μ*^2^〉 < 0.1 Å^2^)) at *T_β_* of their own in CuZr_2_ and Cu_46_Zr_46_Al_8_ MGs.

**
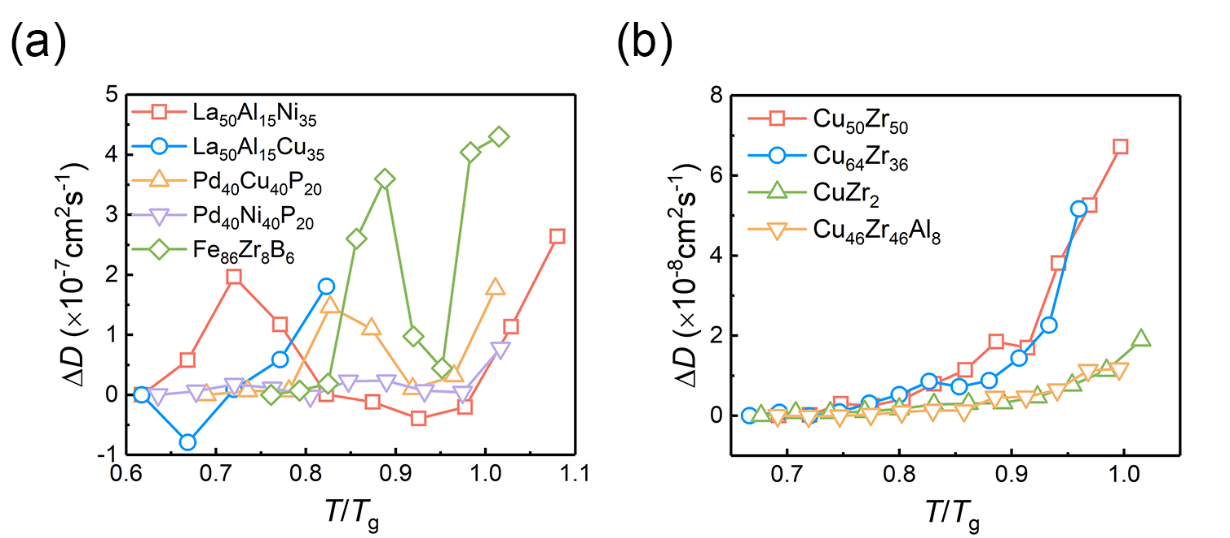
**

**Figure S13.** Temperature-dependent diffusion coefficient change (Δ*D* = *D_i_* - *D*_0_) for all the studied MGs.


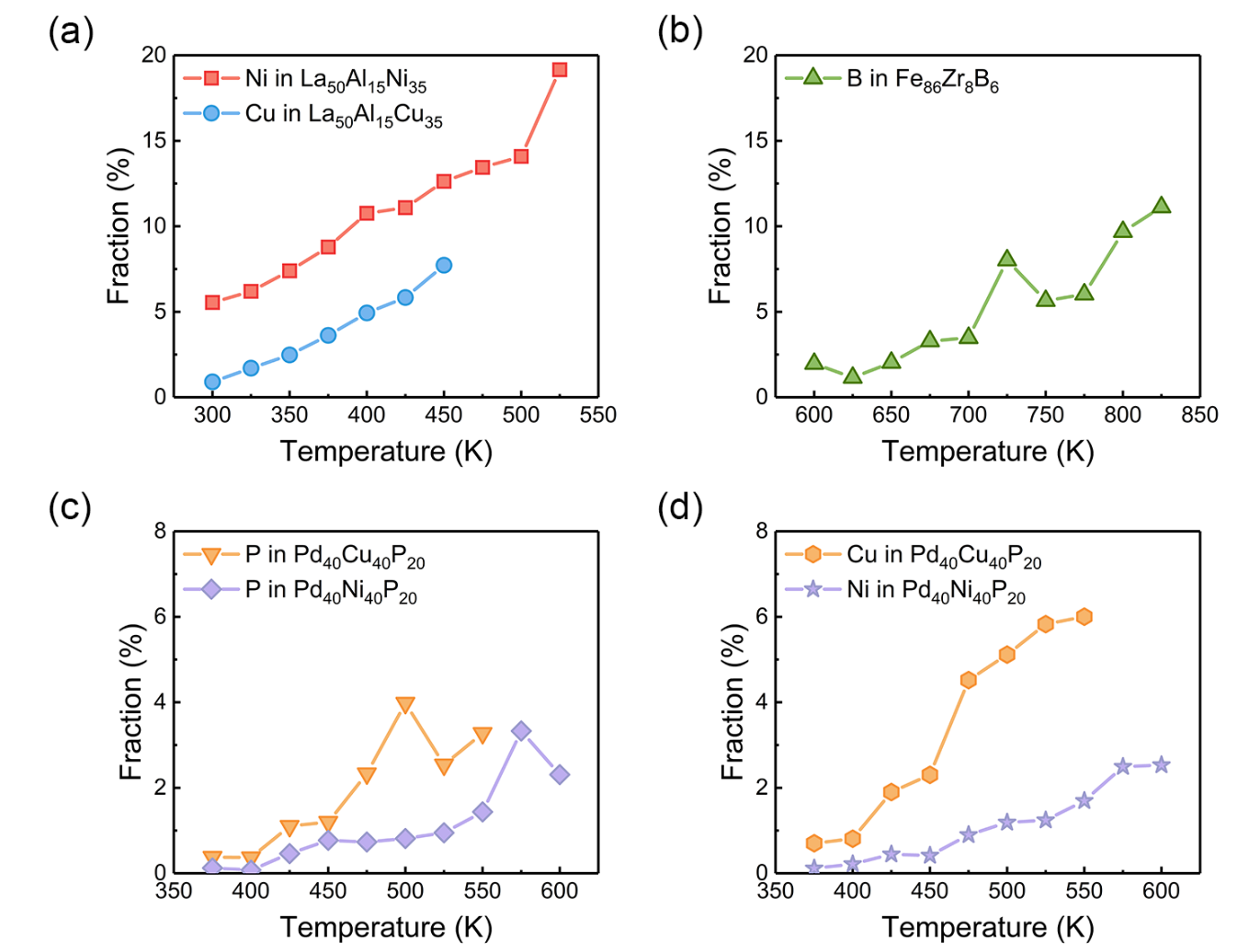


**Figure S14.** Percentage of fast atoms (〈*μ*^2^〉 > 0.5 Å^2^) as a function of temperature in 8,000-step configurations. (a) Ni in La_50_Al_15_Ni_35_ MG and Cu in La_50_Al_15_Cu_35_ MG; (b) B in Fe_86_Zr_8_B_6_ MG; (c) P in both Pd_40_Cu_40_P_20_ and Pd_40_Ni_40_P_20_ MGs; (d) Cu in Pd_40_Cu_40_P_20_ MG and Ni in Pd_40_Ni_40_P_20_ MG. Percentages are calculated relative to the total number of selected atoms.


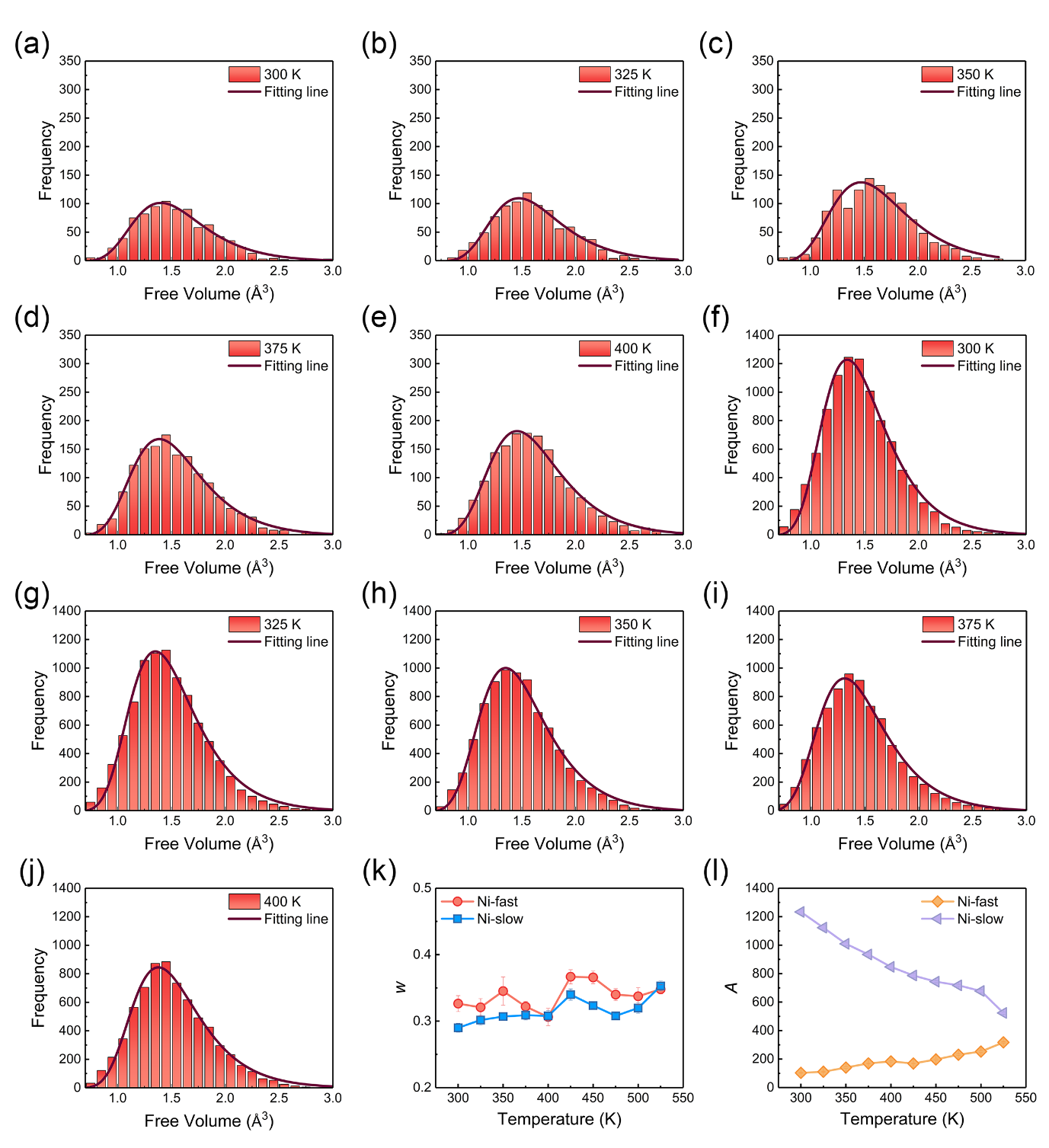


**Figure S15.** Gumbel fitting analysis of free volume distribution for fast and slow Ni atoms in the La_50_Al_15_Ni_35_ MG. The result of the Gumbel function fitting for distribution of free volumes for fast Ni atoms at (a) 300 K; (b) 325 K; (c) 350 K; (d) 375 K; (e) 400 K and slow Ni atoms at (f) 300 K; (g) 325 K; (h) 350 K; (i) 375 K; (j) 400 K in the La_50_Al_15_Ni_35_ MG. Value of (k) *w* and (l) *A* of both fast and slow Ni atoms in the La_50_Al_15_Ni_35_ MG in Gumbel function evolve with heating. Error bar indicates the standard error of the *w* and *A* in the Gumbel fit to the free volume distribution.

**
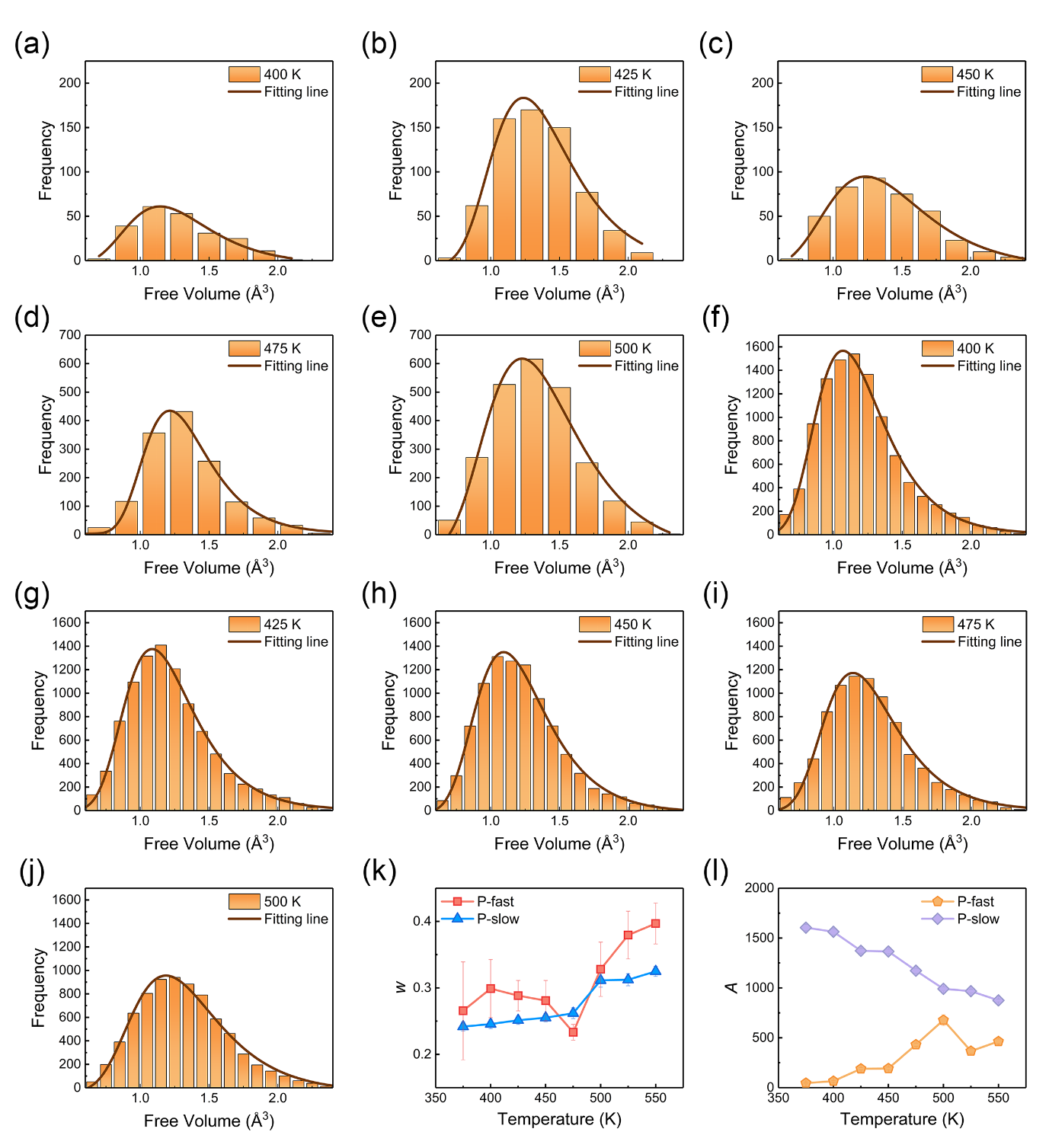
**

**Figure S16.** Gumbel fitting analysis of free volume distribution for fast and slow P atoms in the Pd_40_Cu_40_P_20_ MG. The result of the Gumbel function fitting for distribution of free volumes for fast P atoms at (a) 400 K; (b) 425 K; (c) 450 K; (d) 475 K; (e) 500 K and slow P atoms at (f) 400 K; (g) 425 K; (h) 450 K; (i) 475 K; (j) 500 K in the Pd_40_Cu_40_P_20_ MG. Value of (k) *w* and (l) *A* of both fast and slow P atoms in the Pd_40_Cu_40_P_20_ MG in Gumbel function evolve with heating. Error bar indicates the standard error of the *w* and *A* in the Gumbel fit to the free volume distribution.

**
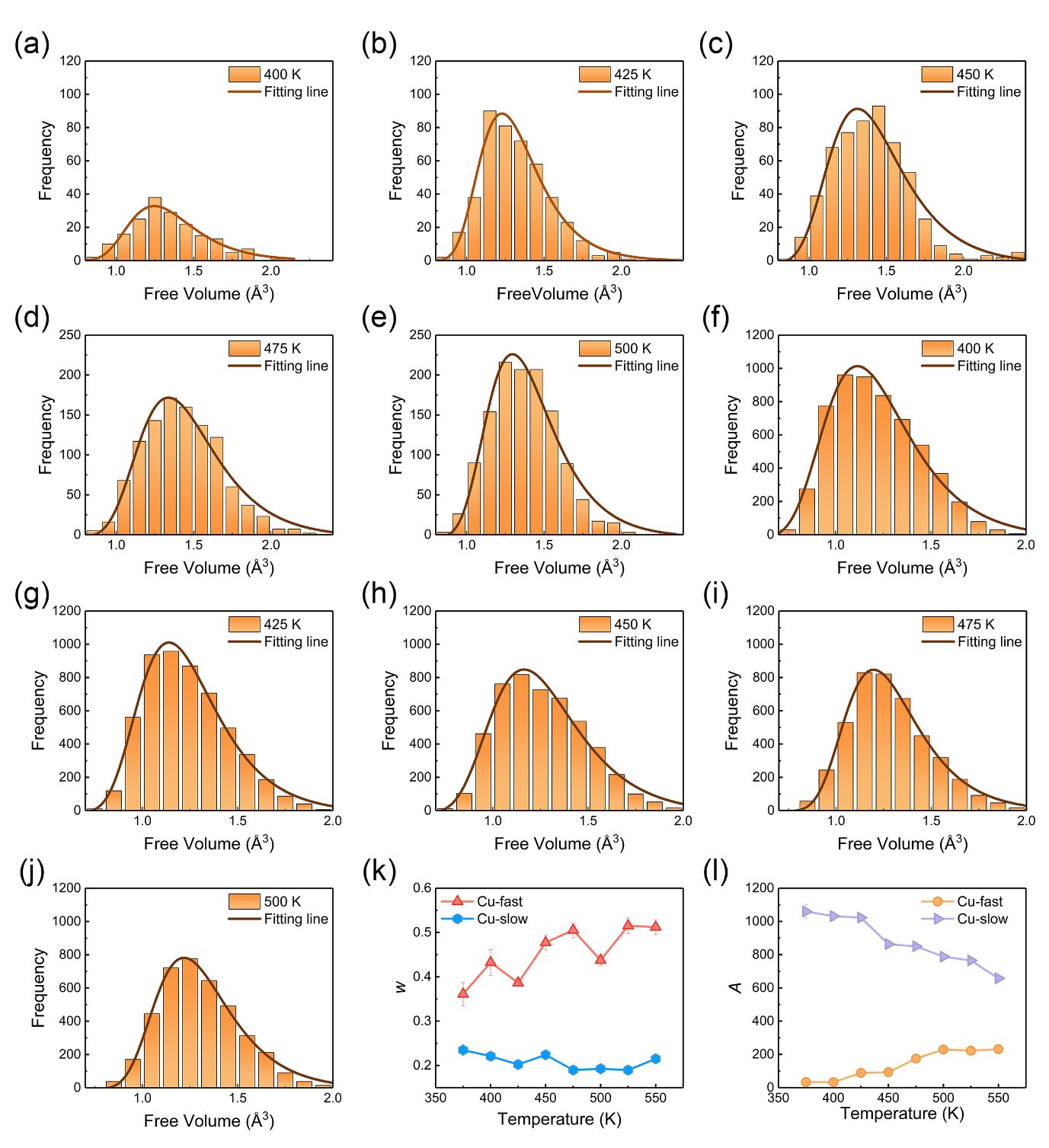
**

**Figure S17.** Gumbel fitting analysis of free volume distribution for fast and slow Cu atoms in the Pd_40_Cu_40_P_20_ MG. The result of the Gumbel function fitting for distribution of free volumes for fast Cu atoms at (a) 400 K; (b) 425 K; (c) 450 K; (d) 475 K; (e) 500 K and slow Cu atoms at (f) 400 K; (g) 425 K; (h) 450 K; (i) 475 K; (j) 500 K in the Pd_40_Cu_40_P_20_ MG. Value of (k) *w* and (l) *A* of both fast and slow Cu atoms in the Pd_40_Cu_40_P_20_ MG in Gumbel function evolve with heating. Error bar indicates the standard error of the *w* and *A* in the Gumbel fit to the free volume distribution.

**
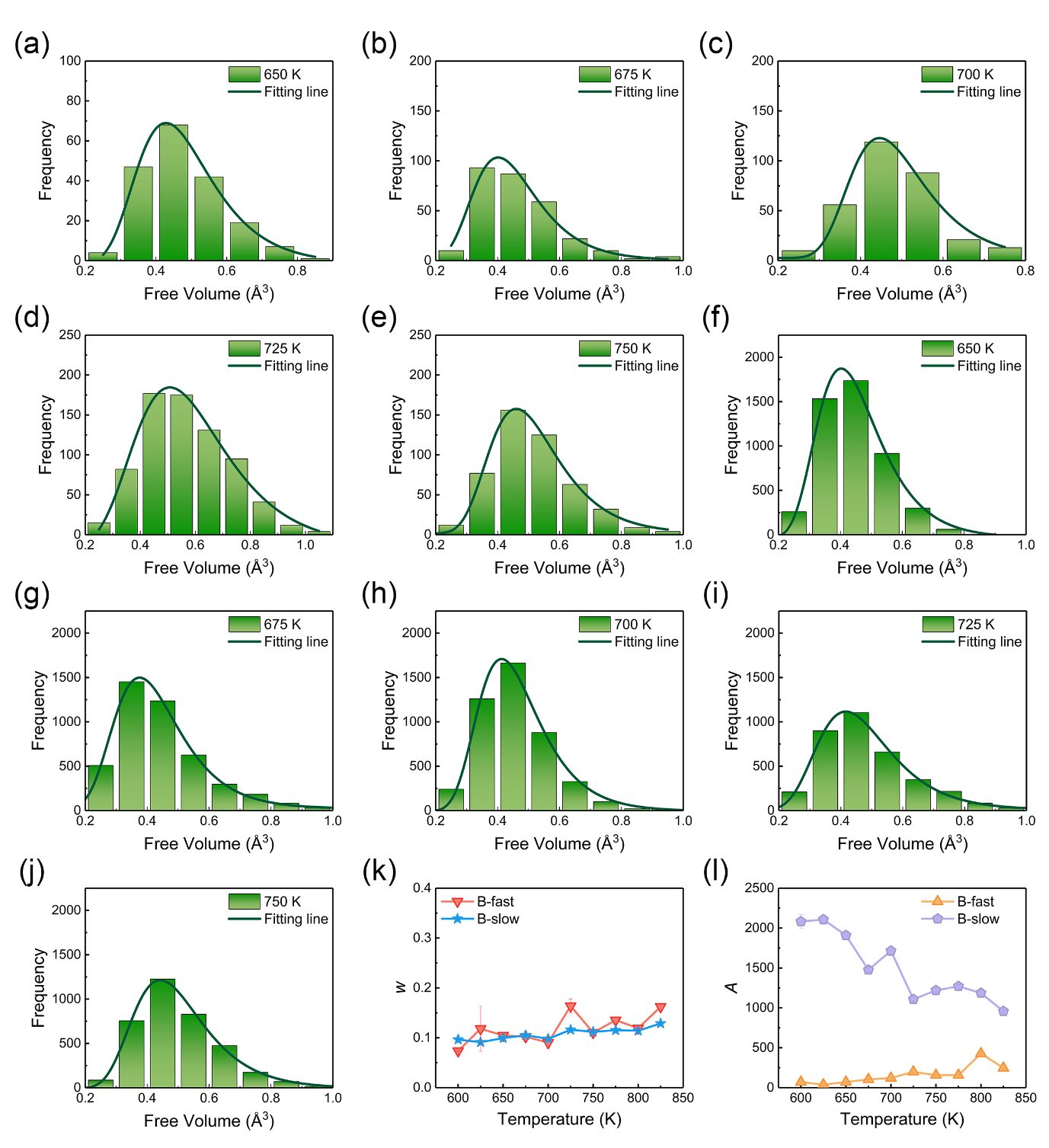
**

**Figure S18.** Gumbel fitting analysis of free volume distribution for fast and slow B atoms in the Fe_86_Zr_8_B_6_ MG. The result of the Gumbel function fitting for distribution of free volumes for fast B atoms at (a) 650 K; (b) 675 K; (c) 700 K; (d) 725 K; (e) 750 K and slow B atoms at (f) 650 K; (g) 675 K; (h) 700 K; (i) 725 K; (j) 750 K in the Fe_86_Zr_8_B_6_ MG. Value of (k) *w* and (l) *A* of both fast and slow B atoms in the Fe_86_Zr_8_B_6_ MG in Gumbel function evolve with heating. Error bar indicates the standard error of the *w* and *A* in the Gumbel fit to the free volume distribution.

**
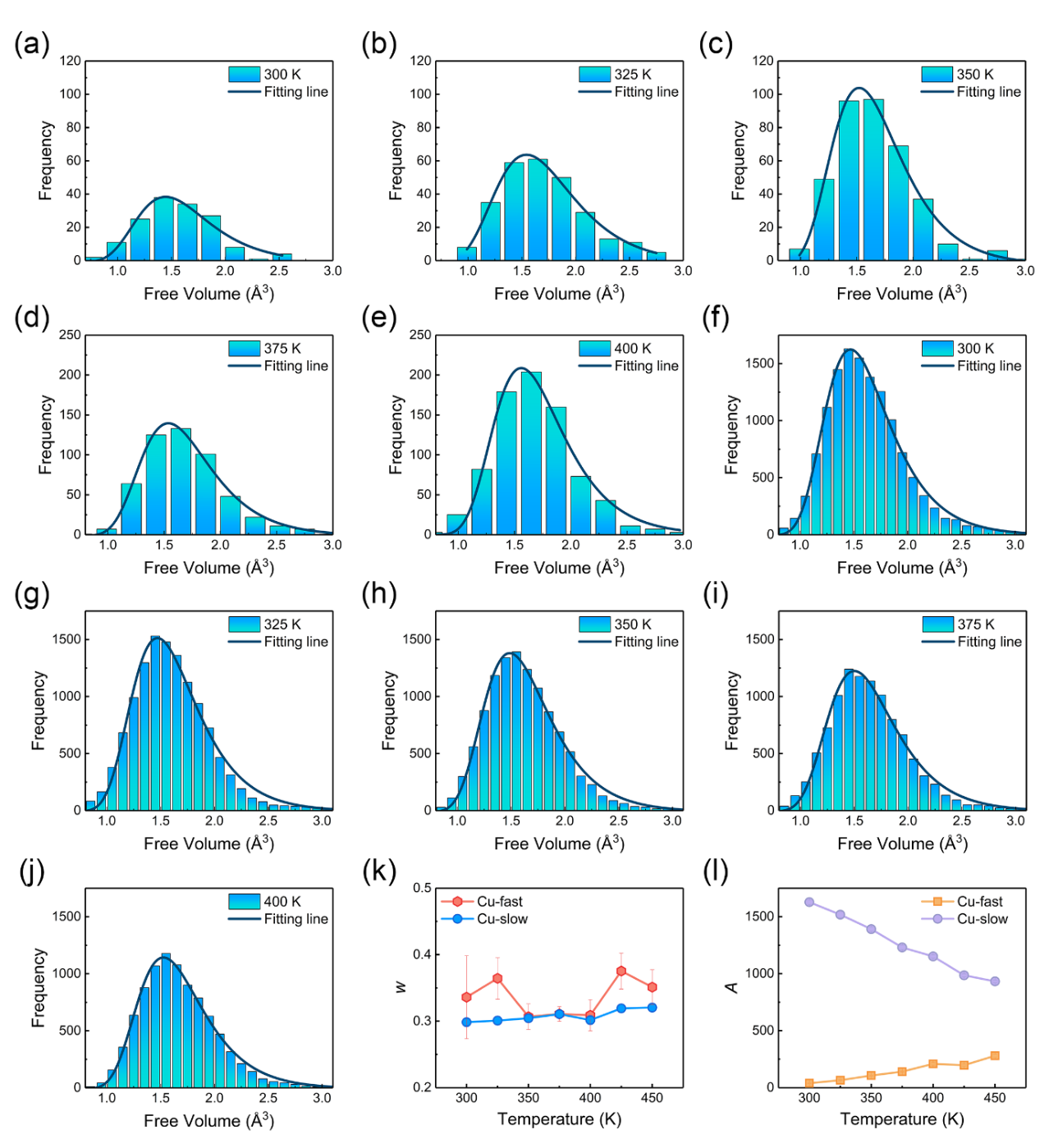
**

**Figure S19.** Gumbel fitting analysis of free volume distribution for fast and slow Cu atoms in the La_50_Al_15_Cu_35_ MG. The result of the Gumbel function fitting for distribution of free volumes for fast Cu atoms at (a) 300 K; **(**b) 325 K; (c) 350 K; (d) 375 K; (e) 400 K and slow Cu atoms at (f) 300 K; (g) 325 K; (h) 350 K; (i) 375 K; (j) 400 K in the La_50_Al_15_Cu_35_ MG. Value of (k) *w* and (l) *A* of both fast and slow Cu atoms in the La_50_Al_15_Cu_35_ MG in Gumbel function evolve with heating. Error bar indicates the standard error of the *w* and *A* in the Gumbel fit to the free volume distribution.

**
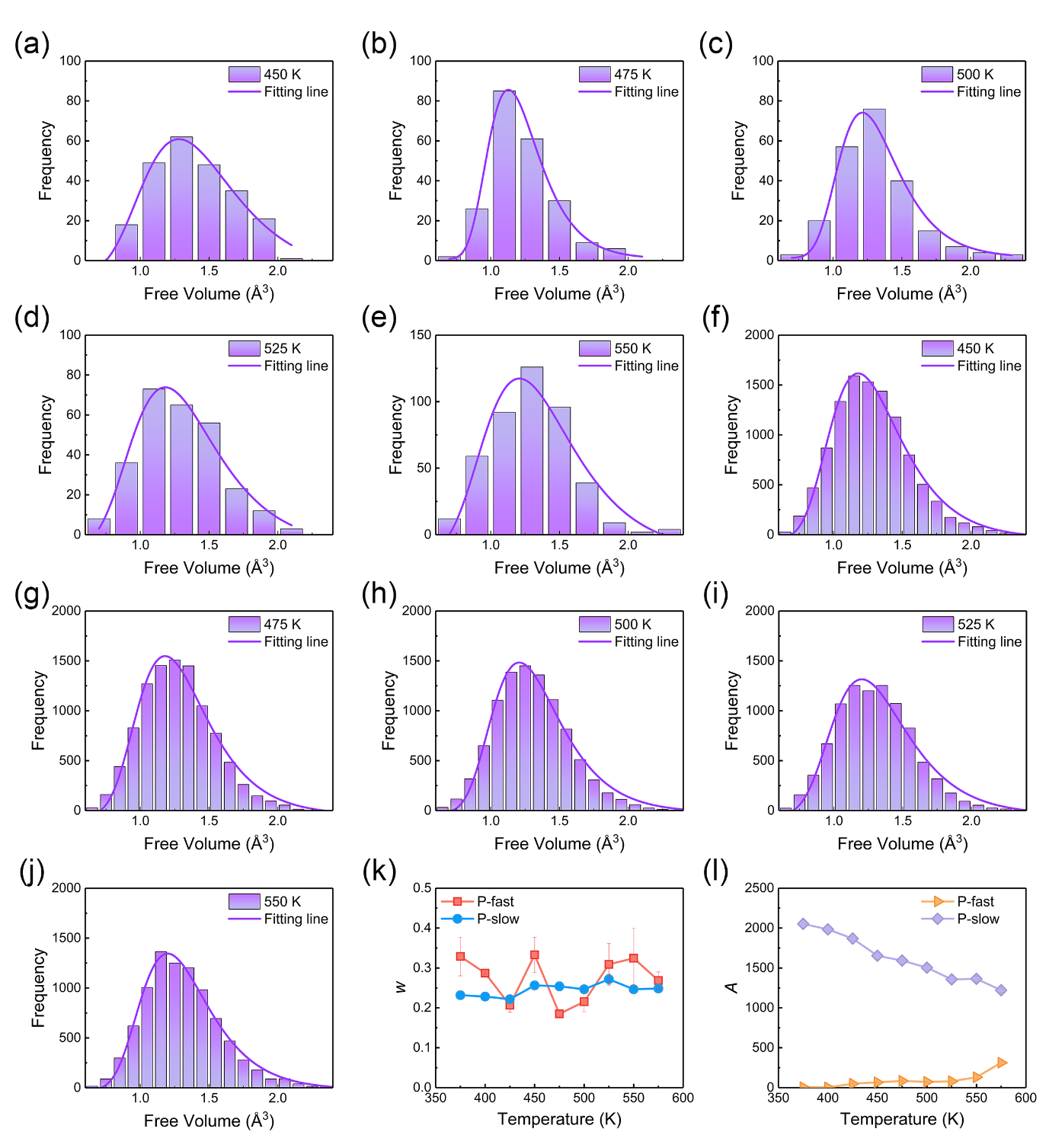
**

**Figure S20.** Gumbel fitting analysis of free volume distribution for fast and slow P atoms in the Pd_40_Ni_40_P_20_ MG. The result of the Gumbel function fitting for distribution of free volumes for fast P atoms at (a) 450 K; (b) 475 K; (c) 500 K; (d) 525 K; (e) 550 K and slow P atoms at (f) 450 K; (g) 475 K; (h) 500 K; (i) 525 K; (j) 550 K in the Pd_40_Ni_40_P_20_ MG. Value of (k) *w* and (l) *A* of both fast and slow P atoms in the Pd_40_Ni_40_P_20_ MG in Gumbel function evolve with heating. Error bar indicates the standard error of the *w* and *A* in the Gumbel fit to the free volume distribution.


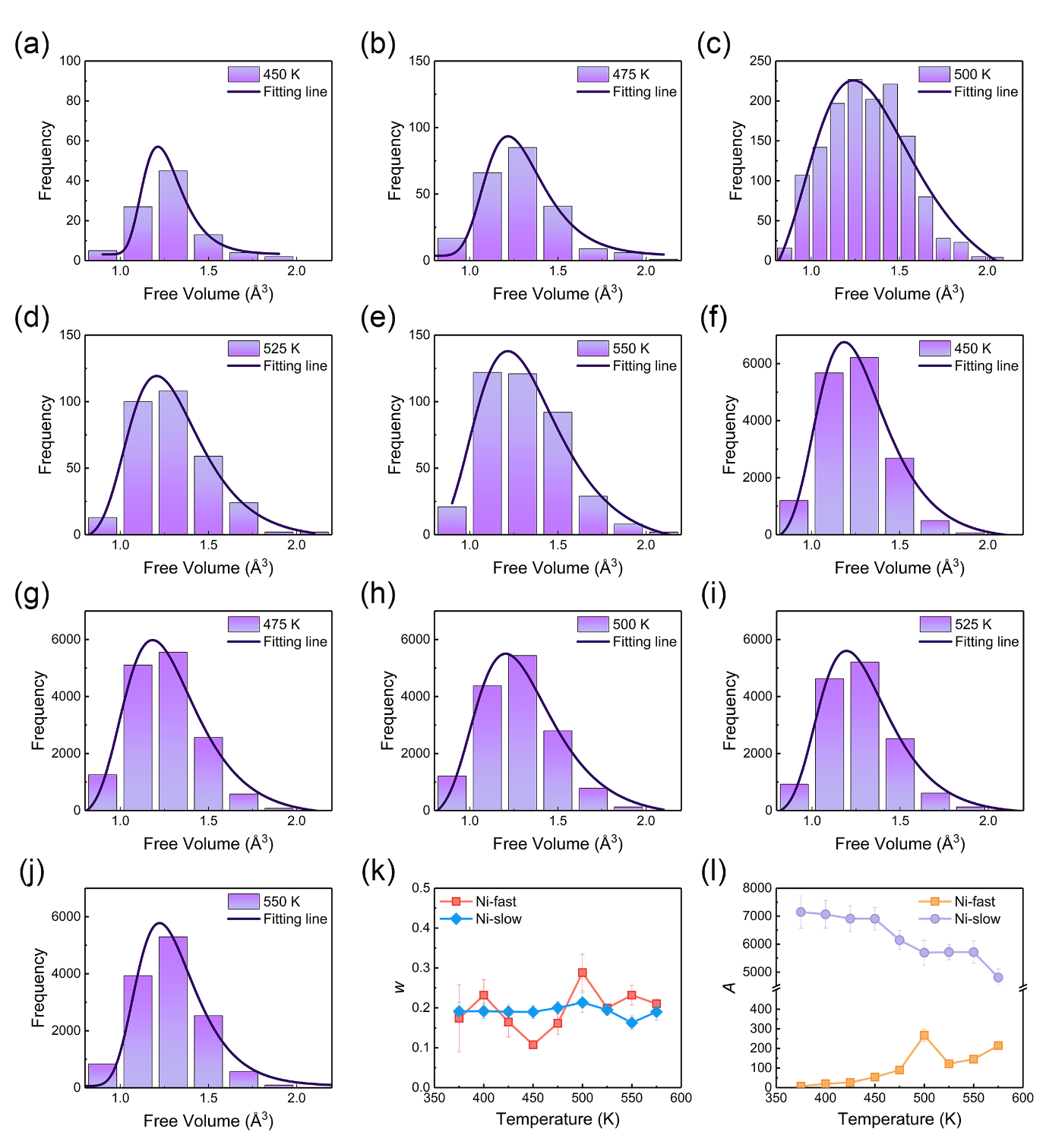


**Figure S21.** Gumbel fitting analysis of free volume distribution for fast and slow Ni atoms in the Pd_40_Ni_40_P_20_ MG. The result of the Gumbel function fitting for distribution of free volumes for fast Ni atoms at (a) 450 K; (b) 475 K; (c) 500 K; (d) 525 K; (e) 550 K and slow Ni atoms at (f) 450 K; (g) 475 K; (h) 500 K; (i) 525 K; (j) 550 K in the Pd_40_Ni_40_P_20_ MG. Value of (k) *w* and (l) *A* of both fast and slow Ni atoms in the Pd_40_Ni_40_P_20_ MG in Gumbel function evolve with heating. Error bar indicates the standard error of the *w* and *A* in the Gumbel fit to the free volume distribution.


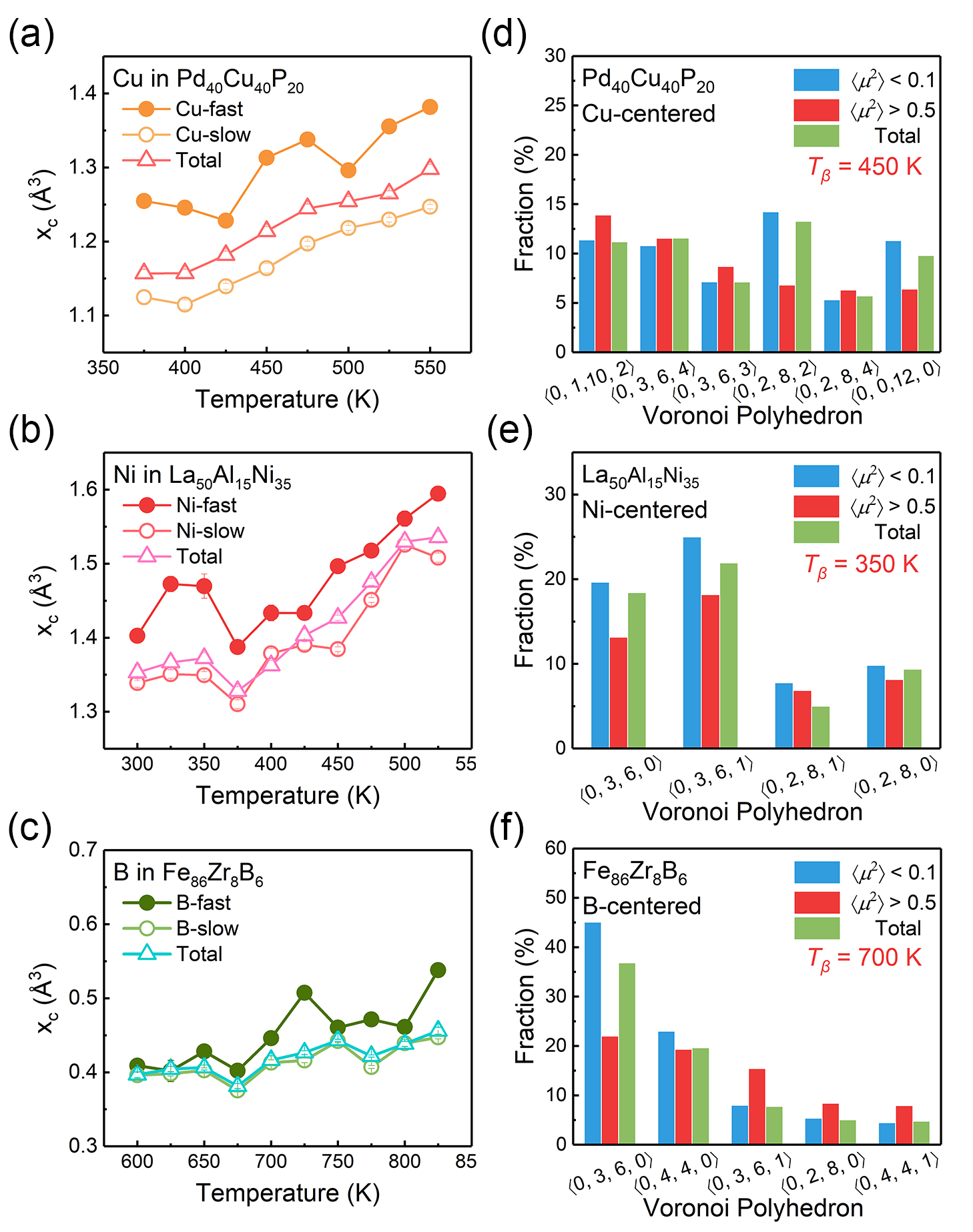


**Figure S22.** Comparison of Voronoi polyhedra and free volume between the total system and partial (fast/slow) atoms with pronounced *β-*relaxation. Voronoi polyhedra of the total atoms and fast/slow atoms in (a) Cu atoms within Pd_40_Cu_40_P_20_, (b) Ni atoms within La_50_Al_15_Ni_35_ and (c) B atoms within Fe_86_Zr_8_B_6_ MGs. Free volume of the total atoms and fast/slow atoms in (d) Cu atoms within Pd_40_Cu_40_P_20_, (e) Ni atoms within La_50_Al_15_Ni_35_ and (f) B atoms within Fe_86_Zr_8_B_6_ MGs.

**
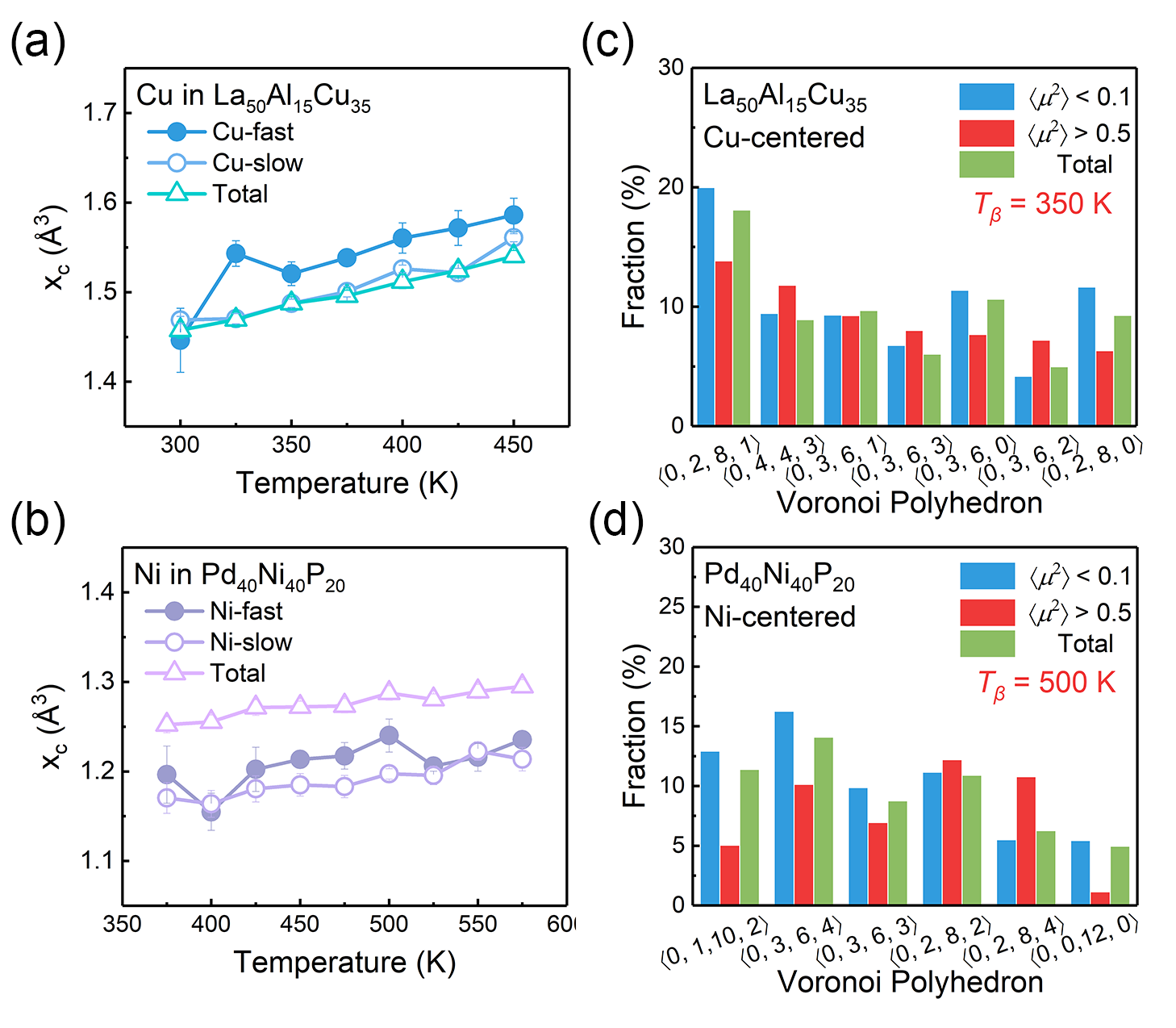
**

**Figure S23.** Comparison of Voronoi polyhedra and free volume between the total system and partial (fast/slow) atoms in systems without pronounced *β-*relaxation. Voronoi polyhedra of the total atoms and fast/slow atoms in (a) Cu atoms within La_50_Al_15_Cu_35_, (b) Ni atoms within Pd_40_Ni_40_P_20_ MGs. Free volume of the total atoms and fast/slow atoms in (d) Cu atoms within La_50_Al_15_Cu_35_, (e) Ni atoms within Pd_40_Ni_40_P_20_ MGs.


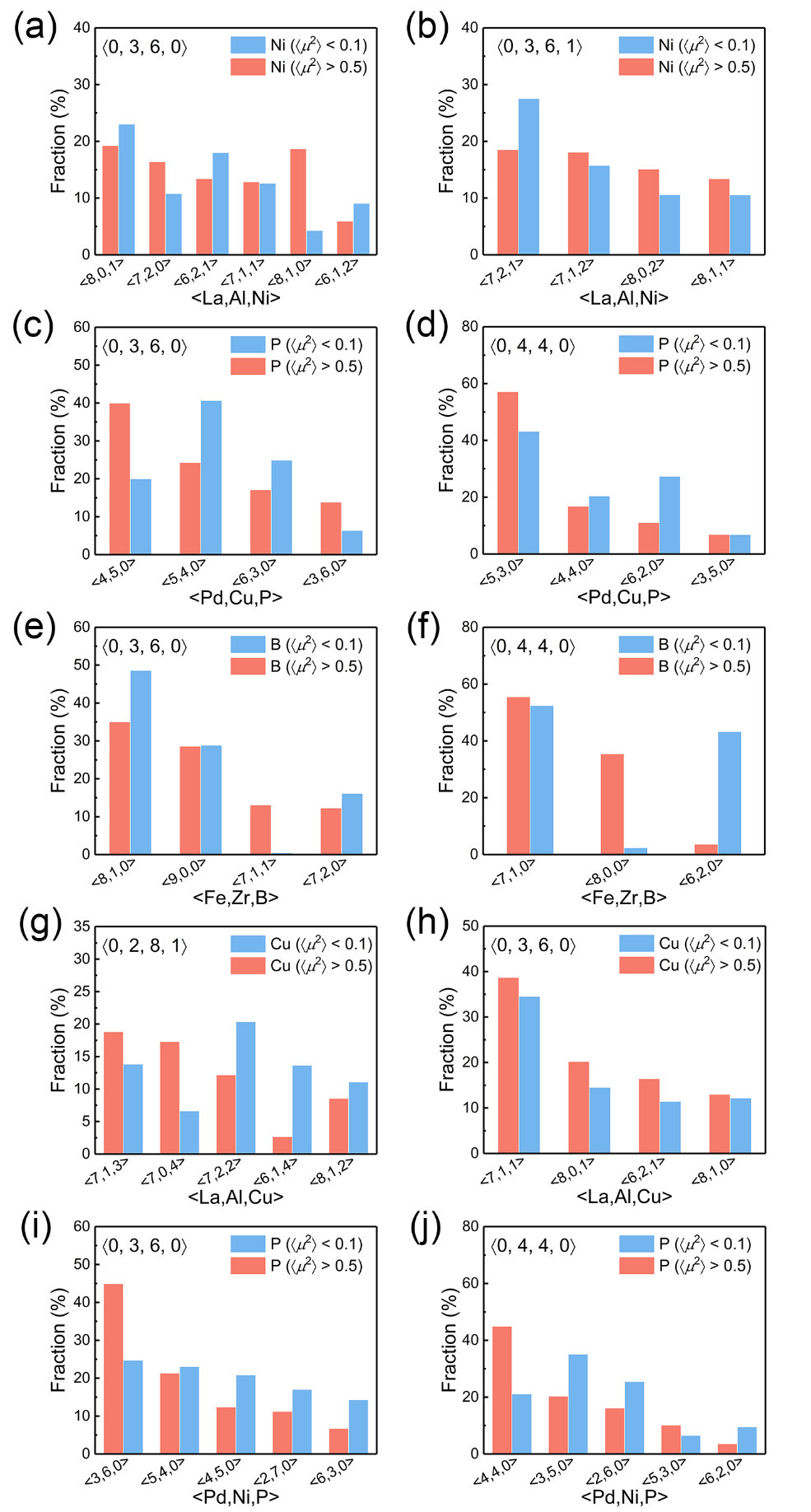


**Figure S24.** Fractions of major polyhedra centered by selected slow and fast atoms with different elemental constitutions in five studied MGs at their *T_β_* temperatures. (a) and (b) La_50_Al_15_Ni_35_, (c) and (d) Pd_40_Cu_40_P_20_, (e) and (f) Fe_86_Zr_8_B_6_, (g) and (h) La_50_Al_15_Cu_35_ and (i) and (j) Pd_40_Ni_40_P_20_ MGs.


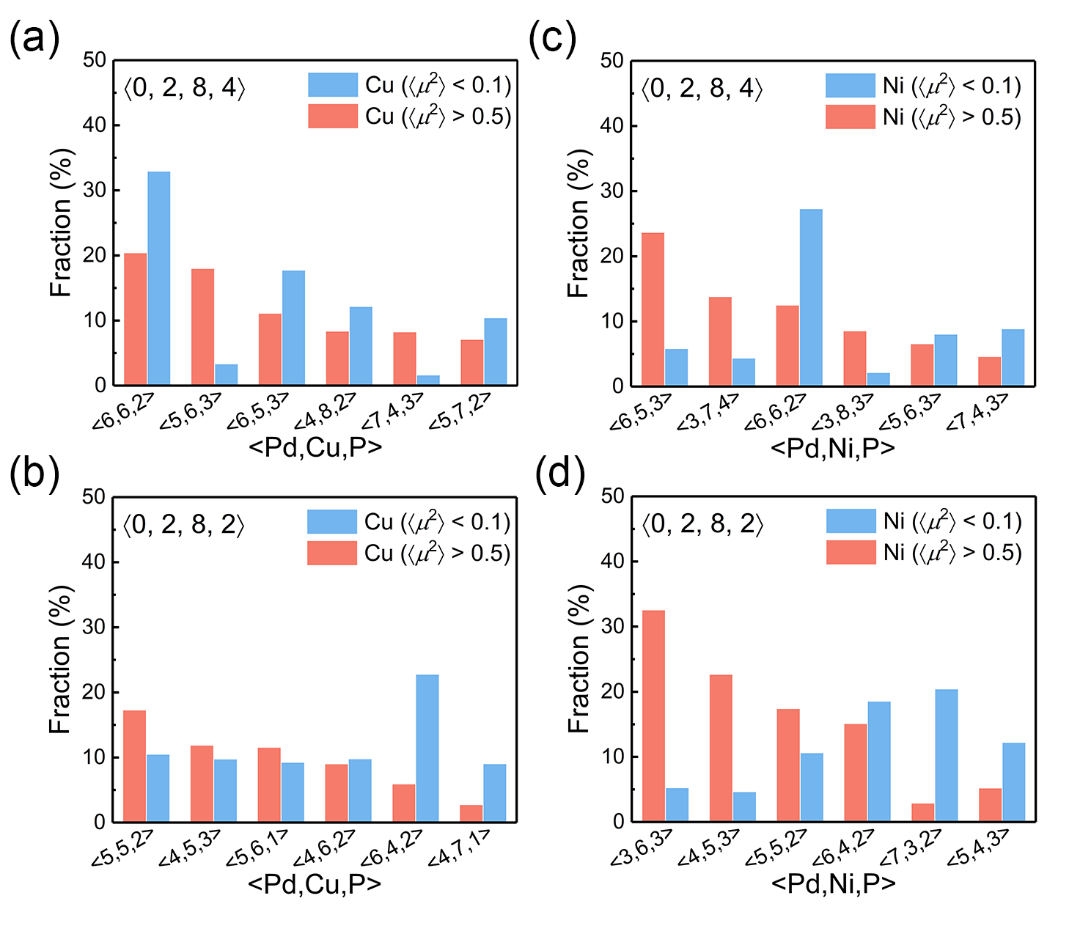


**Figure S25.** Partial CN of major VPs of atoms in Pd_40_Cu_40_P_20_ and Pd_40_Ni_40_P_20_ MGs. Fractions of partial CN of (a) VP 〈0, 2, 8, 4〉 and (b) VP 〈0, 2, 8, 2〉 of Cu atoms in Pd_40_Cu_40_P_20_ MG. Fractions of partial CN of (c) VP 〈0, 2, 8, 4〉 and (d) VP 〈0, 2, 8, 2〉 of Ni atoms in Pd_40_Ni_40_P_20_ MG.


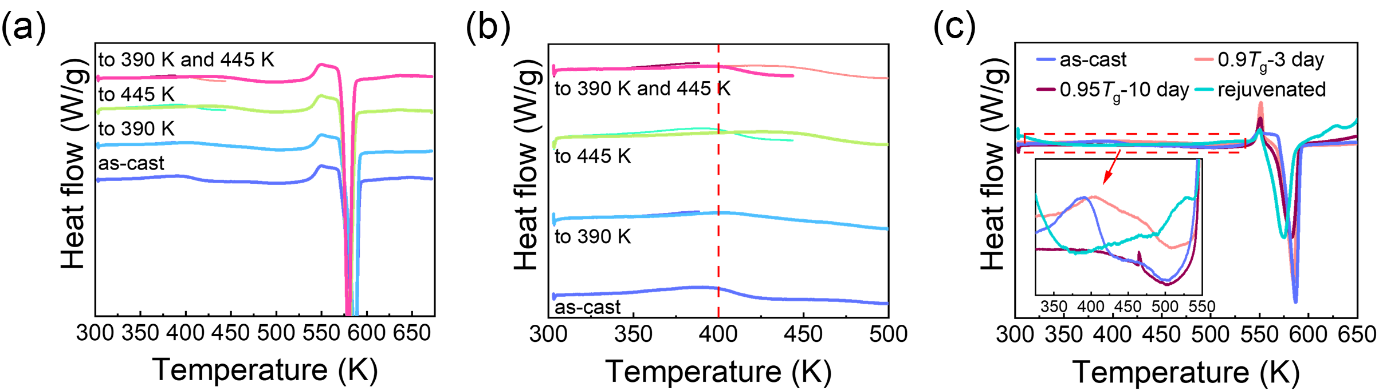


**Figure S26.** Evolution of DSC profiles for Pd_40_Cu_40_P_20_ MG during cyclic heating. (a) The temperature dependence of heat flow under different heating profiles at 10 K/min. (b) An enlargement of heat flow curves below 500 K. (c) Comparisons of heat flows for four studied samples under different thermal histories. Inset shows the local magnification of four exothermic peaks below *T*_g_.


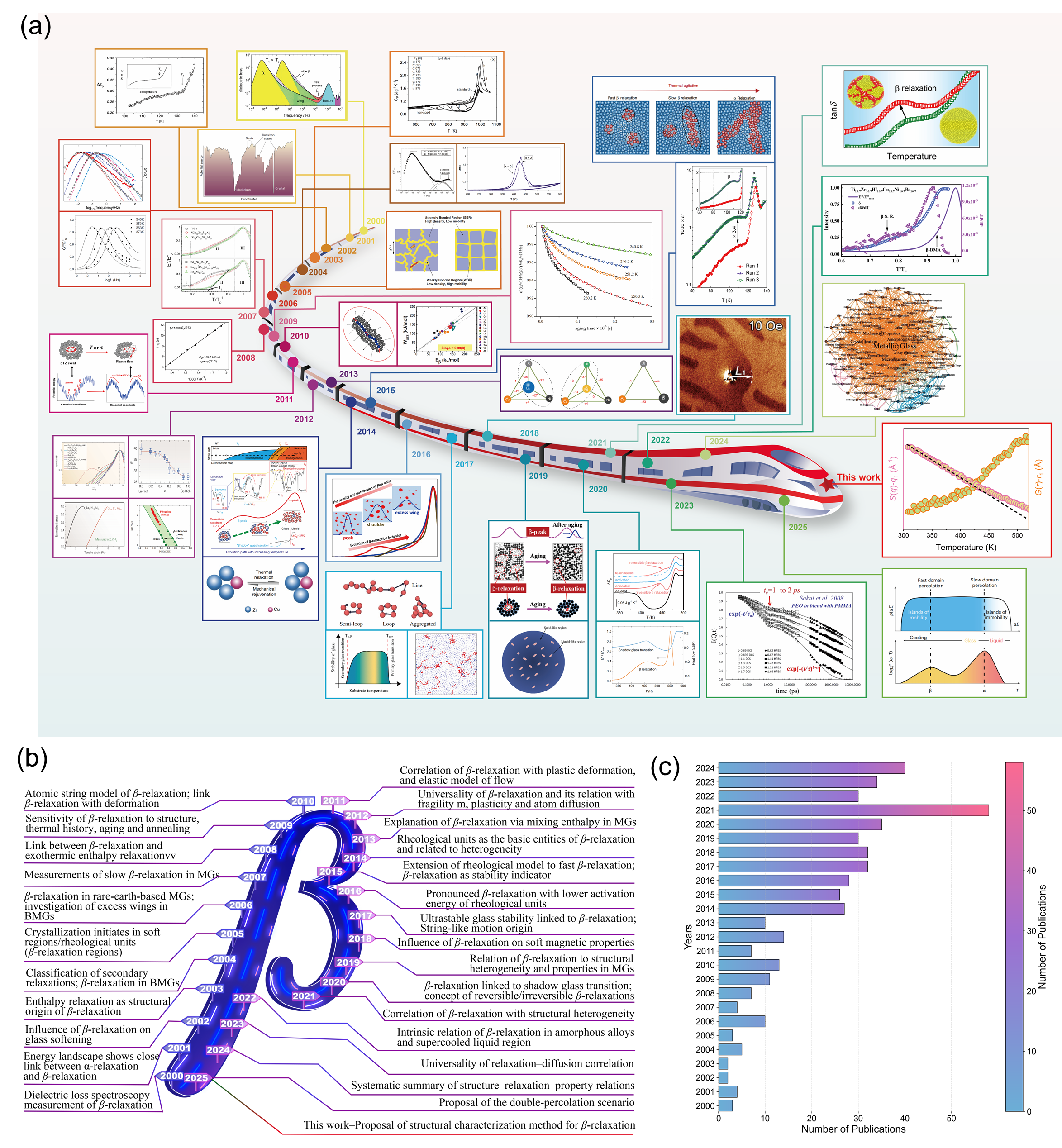


**Figure S27.** (a) Developments in *β*-relaxation of metallic glasses over the past 25 years [1-55] and the significance of the structural characterization approach proposed in this work to capture *β*-relaxation. Figures within are reproduced with permission from references [1, 2, 5, 7, 8, 11-15, 18-25, 29-31, 34, 36, 38, 41-43, 47-55] with Copyright 2000, Taylor & Francis; Copyright 2001, Springer Nature; Copyright 2002, AIP Publishing; Copyright 2003, IOP Publishing; Copyright 2004, AIP Publishing; Copyright 2004, Elsevier; Copyright 2005, American Physical Society; Copyright 2006, Elsevier; Copyright 2006, Elsevier; Copyright 2007, American Physical Society; Copyright 2008, American Chemical Society; Copyright 2009, American Physical Society; Copyright 2010, Elsevier; Copyright 2010, American Physical Society; Copyright 2011, AIP Publishing; Copyright 2012, American Physical Society; Copyright 2012, Elsevier; Copyright 2012, AIP Publishing; Copyright 2012, American Physical Society; Copyright 2013, Springer Nature; Copyright 2014, Springer Nature; Copyright 2014, Springer Nature; Copyright 2015, Springer Nature; Copyright 2015, American Physical Society; Copyright 2016, AIP Publishing; Copyright 2017, American Association for the Advancement of Science; Copyright 2017, American Chemical Society; Copyright 2018, American Physical Society; Copyright 2019, Elsevier; Copyright 2019, Elsevier; Copyright 2020, American Physical Society; Copyright 2021, American Chemical Society; Copyright 2022, American Physical Society; Copyright 2023, Elsevier; Copyright 2024, Elsevier; Copyright 2025, Springer Nature; (b) The major research findings and (c) the annual number of related publications.

**Table S1.** The coordination numbers (CNs) for five studied MGs at their respective *T_β_*.

| Alloy | | Slow atoms [〈*µ*^2^〉 < 0.1 Å^2^] | | | | Fast atoms [〈*µ*^2^〉 > 0.5 Å^2^] | | | | |
| --- | --- | --- | --- | --- | --- | --- | --- | --- | --- | --- |
|  |  | CNs | | | | | | | | |
|  |  | Total | La | Al | Ni/Cu | | Total | La | Al | Ni/Cu |
| La_50_Al_15_Ni_35_ | La | 15.02 | 7.74 | 2.28 | 5.00 | | 13.41 | 6.62 | 1.99 | 4.80 |
|  | Al | 11.73 | 7.38 | 1.13 | 3.22 | | 10.88 | 6.88 | 0.98 | 3.02 |
|  | Ni | 9.94 | 6.99 | 1.47 | 1.48 | | 9.85 | 7.20 | 1.08 | 1.57 |
| La_50_Al_15_Cu_35_ | La | 14.88 | 7.46 | 2.41 | 5.01 | | 13.45 | 6.97 | 1.84 | 4.64 |
|  | Al | 11.62 | 7.69 | 0.81 | 3.12 | | 11.37 | 7.22 | 0.85 | 3.31 |
|  | Cu | 10.69 | 7.08 | 1.32 | 2.29 | | 10.80 | 7.19 | 1.48 | 2.13 |
|  | | Total | Pd | Cu/Ni | P | | Total | Pd | Cu/Ni | P |
| Pd_40_Cu_40_P_20_ | Pd | 13.95 | 5.63 | 5.54 | 2.78 | | 13.75 | 4.98 | 5.62 | 3.15 |
|  | Cu | 12.75 | 5.48 | 5.16 | 2.11 | | 12.55 | 5.50 | 4.50 | 2.55 |
|  | P | 8.60 | 4.97 | 3.63 | 0 | | 8.26 | 4.79 | 3.47 | 0 |
| Pd_40_Ni_40_P_20_ | Pd | 13.89 | 6.16 | 5.48 | 2.25 | | 13.96 | 5.77 | 5.85 | 2.34 |
|  | Ni | 12.92 | 5.52 | 4.88 | 2.52 | | 13.02 | 4.85 | 5.31 | 2.85 |
|  | P | 8.29 | 3.56 | 4.73 | 0 | | 8.29 | 3.61 | 4.68 | 0 |
|  | | Total | Fe | Zr | B | | Total | Fe | Zr | B |
| Fe_86_Zr_8_B_6_ | Fe | 12.66 | 10.65 | 1.46 | 0.55 | | 12.55 | 10.66 | 1.34 | 0.55 |
|  | Zr | 17.54 | 15.35 | 0.96 | 1.23 | | 16.60 | 14.92 | 1.02 | 0.66 |
|  | B | 8.86 | 7.50 | 1.36 | 0 | | 8. 75 | 7.53 | 1.22 | 0 |

**Table S2.** The average bond lengths in five studied MGs at their respective *T_β_*.

| Alloys | | Slow atoms [〈*µ*^2^〉 < 0.1 Å^2^] | | | | | Fast atoms [〈*µ*^2^〉 > 0.5 Å^2^] | | | | |
| --- | --- | --- | --- | --- | --- | --- | --- | --- | --- | --- | --- |
|  |  | Bond length [Å] | | | | | | | | | |
|  |  | Average | La | Al | Ni/Cu | Total average | Average | La | Al | Ni/Cu | Total average |
| La_50_Al_15_Ni_35_ | La | 3.527 | 3.785 | 3.421 | 3.174 | 3.354 | 3.549 | 3.773 | 3.480 | 3.209 | 3.345 |
|  | Al | 3.164 | 3.432 | 2.896 | 2.646 |  | 3.204 | 3.434 | 2.915 | 2.743 |  |
|  | Ni | 3.068 | 3.179 | 2.630 | 2.981 |  | 3.141 | 3.205 | 2.756 | 3.100 |  |
| La_50_Al_15_Cu_35_ | La | 3.552 | 3.798 | 3.403 | 3.256 | 3.387 | 3.576 | 3.778 | 3.446 | 3.285 | 3.399 |
|  | Al | 3.232 | 3.407 | 3.083 | 2.838 |  | 3.317 | 3.426 | 3.219 | 3.083 |  |
|  | Cu | 3.153 | 3.264 | 2.851 | 2.983 |  | 3.201 | 3.281 | 2.993 | 3.073 |  |
|  | | Average | Pd | Cu/Ni | P | Total average | Average | Pd | Cu/Ni | P | Total average |
| Pd_40_Cu_40_P_20_ | Pd | 2.850 | 2.990 | 2.850 | 2.565 | 2.756 | 2.863 | 2.994 | 2.870 | 2.645 | 2.795 |
|  | Cu | 2.750 | 2.846 | 2.712 | 2.590 |  | 2.805 | 2.900 | 2.779 | 2.626 |  |
|  | P | 2.470 | 2.475 | 2.464 | / |  | 2.469 | 2.471 | 2.466 | / |  |
| Pd_40_Ni_40_P_20_ | Pd | 2.861 | 2.956 | 2.845 | 2.640 | 2.775 | 2.891 | 2.970 | 2.899 | 2.677 | 2.746 |
|  | Ni | 2.708 | 2.847 | 2.725 | 2.372 |  | 2.736 | 2.900 | 2.744 | 2.442 |  |
|  | P | 2.384 | 2.488 | 2.307 | / |  | 2.413 | 2.499 | 2.347 | / |  |
|  | | Average | Fe | Zr | B | Total average | Average | Fe | Zr | B | Total average |
| Fe_86_Zr_8_B_6_ | Fe | 2.558 | 2.525 | 2.920 | 2.240 | 2.599 | 2.573 | 2.549 | 2.898 | 2.251 | 2.565 |
|  | Zr | 2.914 | 2.912 | 3.249 | 2.681 |  | 2.929 | 2.913 | 3.278 | 2.728 |  |
|  | B | 2.253 | 2.187 | 2.616 | / |  | 2.274 | 2.223 | 2.602 | / |  |

**References**

[1] P. Lunkenheimer, U. Schneider, R. Brand, A. Loid, Glassy dynamics, Contemporary Physics 41(1) (2000) 15-36.

[2] P.G. Debenedetti, F.H. Stillinger, Supercooled liquids and the glass transition, Nature 410(6825) (2001) 259-267.

[3] T.C. Hufnagel, C.A. Schuh, M.L. Falk, Deformation of metallic glasses: Recent developments in theory, simulations, and experiments, Acta Materialia 109 (2016) 375-393.

[4] Y. Cheng, E. Ma, Atomic-level structure and structure–property relationship in metallic glasses, Progress in Materials Science 56(4) (2011) 379-473.

[5] G. Johari, G. Power, J. Vij, Localized relaxation’s strength and its mimicry of glass-softening thermodynamics, The Journal of Chemical Physics 116(13) (2002) 5908-5909.

[6] G. Johari, Localized molecular motions of *β*-relaxation and its energy landscape, Journal of Non-crystalline solids 307 (2002) 317-325.

[7] C.A. Angell, Y. Yue, L.-M. Wang, J.R. Copley, S. Borick, S. Mossa, Potential energy, relaxation, vibrational dynamics and the boson peak, of hyperquenched glasses, Journal of Physics: Condensed Matter 15(11) (2003) S1051.

[8] K. Ngai, M. Paluch, Classification of secondary relaxation in glass-formers based on dynamic properties, The Journal of Chemical Physics 120(2) (2004) 857-873.

[9] H. Tanaka, Origin of the excess wing and slow *β* relaxation of glass formers: A unified picture of local orientational fluctuations, Physical Review E 69(2) (2004) 021502.

[10] Q. Wang, J.-M. Pelletier, Y. Dong, Y. Ji, Structural relaxation and crystallisation of bulk metallic glasses Zr41Ti14Cu12.5Ni10-xBe22.5Fex (x= 0 or 2) studied by mechanical spectroscopy, Materials Science and Engineering: A 370(1-2) (2004) 316-320.

[11] T. Ichitsubo, E. Matsubara, T. Yamamoto, H. Chen, N. Nishiyama, J. Saida, K. Anazawa, Microstructure of fragile metallic glasses inferred from ultrasound-accelerated crystallization in Pd-based metallic glasses, Physical Review Letters 95(24) (2005) 245501.

[12] X. Liu, B. Zhang, P. Wen, W. Wang, The slow *β*-relaxation observed in Ce-based bulk metallic glass-forming supercooled liquid, Journal of Non-crystalline solids 352(38-39) (2006) 4013-4016.

[13] W.H. Wang, P. Wen, X.F. Liu, The excess wing of bulk metallic glass forming liquids, Journal of Non-crystalline solids 352(42-49) (2006) 5103-5109.

[14] Z. Zhao, P. Wen, C. Shek, W. Wang, Measurements of slow *β*-relaxations in metallic glasses and supercooled liquids, Physical Review B 75(17) (2007) 174201.

[15] L. Hu, Y. Yue, Secondary relaxation behavior in a strong glass, The Journal of Physical Chemistry B 112(30) (2008) 9053-9057.

[16] L. Hu, Y. Yue, Secondary relaxation in metallic glass formers: Its correlation with the genuine Johari− Goldstein relaxation, The Journal of Physical Chemistry C 113(33) (2009) 15001-15006.

[17] C. Zhou, Y. Yue, L. Hu, Revealing the connection between the slow *β* relaxation and sub-Tg enthalpy relaxation in metallic glasses, Journal of Applied Physics 120(22) (2016).

[18] R. Casalini, C. Roland, Aging of the secondary relaxation to probe structural relaxation in the glassy state, Physical Review Letters 102(3) (2009) 035701.

[19] D. Bedorf, K. Samwer, Length scale effects on relaxations in metallic glasses, Journal of Non-crystalline solids 356(6-8) (2010) 340-343.

[20] H. Yu, W. Wang, H. Bai, Y. Wu, M. Chen, Relating activation of shear transformation zones to *β* relaxations in metallic glasses, Physical Review B 81(22) (2010) 220201.

[21] W.H. Wang, Correlation between relaxations and plastic deformation, and elastic model of flow in metallic glasses and glass-forming liquids, Journal of Applied Physics 110(5) (2011).

[22] H. Yu, Z. Wang, W. Wang, H. Bai, Relation between *β* relaxation and fragility in LaCe-based metallic glasses, Journal of Non-crystalline solids 358(4) (2012) 869-871.

[23] Z. Wang, P. Wen, L. Huo, H. Bai, W. Wang, Signature of viscous flow units in apparent elastic regime of metallic glasses, Applied Physics Letters 101(12) (2012).

[24] H.-B. Yu, K. Samwer, Y. Wu, W.H. Wang, Correlation between *β* Relaxation and Self-Diffusion of the Smallest Constituting Atoms in Metallic Glasses, Physical Review Letters 109(9) (2012) 095508.

[25] H. Yu, X. Shen, Z. Wang, L. Gu, W. Wang, H. Bai, Tensile plasticity in metallic glasses with pronounced *β* relaxations, Physical Review Letters 108(1) (2012) 015504.

[26] Y. Cohen, S. Karmakar, I. Procaccia, K. Samwer, The nature of the *β*-peak in the loss modulus of amorphous solids, Europhysics Letters 100(3) (2012) 36003.

[27] J. Qiao, J.-M. Pelletier, Dynamic mechanical analysis in La-based bulk metallic glasses: Secondary (*β*) and main (*α*) relaxations, Journal of Applied Physics 112(8) (2012).

[28] J. Qiao, J.-M. Pelletier, R. Casalini, Relaxation of bulk metallic glasses studied by mechanical spectroscopy, The Journal of Physical Chemistry B 117(43) (2013) 13658-13666.

[29] H.B. Yu, K. Samwer, W.H. Wang, H.Y. Bai, Chemical influence on *β*-relaxations and the formation of molecule-like metallic glasses, Nature Communications 4(1) (2013) 2204.

[30] Z. Wang, B. Sun, H. Bai, W. Wang, Evolution of hidden localized flow during glass-to-liquid transition in metallic glass, Nature Communications 5(1) (2014) 5823.

[31] Y. Liu, T. Fujita, D. Aji, M. Matsuura, M. Chen, Structural origins of Johari-Goldstein relaxation in a metallic glass, Nature Communications 5(1) (2014) 3238.

[32] H.B. Yu, W.H. Wang, H.Y. Bai, K. Samwer, The *β*-relaxation in metallic glasses, National Science Review 1(3) (2014) 429-461.

[33] L. Zhao, W. Wang, H. Bai, Modulation of *β*-relaxation by modifying structural configurations in metallic glasses, Journal of Non-crystalline solids 405 (2014) 207-210.

[34] Q. Wang, S. Zhang, Y. Yang, Y. Dong, C. Liu, J. Lu, Unusual fast secondary relaxation in metallic glass, Nature Communications 6(1) (2015) 7876.

[35] Z. Zhu, Z. Wang, W. Wang, Binary rare earth element-Ni/Co metallic glasses with distinct *β*-relaxation behaviors, Journal of Applied Physics 118(15) (2015).

[36] H. Yu, M. Tylinski, A. Guiseppi-Elie, M. Ediger, R. Richert, Suppression of *β* relaxation in vapor-deposited ultrastable glasses, Physical Review Letters 115(18) (2015) 185501.

[37] F. Zhu, H. Nguyen, S. Song, D.P. Aji, A. Hirata, H. Wang, K. Nakajima, M. Chen, Intrinsic correlation between *β*-relaxation and spatial heterogeneity in a metallic glass, Nature Communications 7(1) (2016) 11516.

[38] Z. Lu, B. Shang, Y. Sun, Z. Zhu, P. Guan, W. Wang, H. Bai, Revealing *β*-relaxation mechanism based on energy distribution of flow units in metallic glass, The Journal of Chemical Physics 144(14) (2016).

[39] Q. Wang, J. Liu, Y. Ye, T. Liu, S. Wang, C. Liu, J. Lu, Y. Yang, Universal secondary relaxation and unusual brittle-to-ductile transition in metallic glasses, Materials Today 20(6) (2017) 293-300.

[40] A. Gulzar, L. Zhao, R. Xue, K. Shahzad, D. Zhao, W. Wang, Correlation between flow units and crystallization in metallic glasses, Journal of Non-Crystalline Solids 461 (2017) 61-66.

[41] K. Ngai, L.-M. Wang, H.-B. Yu, Relating ultrastable glass formation to enhanced surface diffusion via the Johari–Goldstein *β*-relaxation in molecular glasses, The Journal of Physical Chemistry Letters 8(12) (2017) 2739-2744.

[42] H.-B. Yu, R. Richert, K. Samwer, Structural rearrangements governing Johari-Goldstein relaxations in metallic glasses, Science Advances 3(11) (2017) e1701577.

[43] S. Ouyang, L. Song, Y. Liu, J. Huo, J. Wang, W. Xu, J. Li, C. Wang, X. Wang, R. Li, Correlation between the viscoelastic heterogeneity and the domain wall motion of Fe-based metallic glass, Physical Review Materials 2(6) (2018) 063601.

[44] L. Song, W. Xu, J. Huo, J.-Q. Wang, X. Wang, R. Li, Two-step relaxations in metallic glasses during isothermal annealing, Intermetallics 93 (2018) 101-105.

[45] N. He, L. Song, W. Xu, J. Huo, J.-Q. Wang, R.-W. Li, The evolution of relaxation modes during isothermal annealing and its influence on properties of Fe-based metallic glass, Journal of Non-Crystalline Solids 509 (2019) 95-98.

[46] L. Song, M. Gao, W. Xu, J. Huo, J. Wang, R. Li, W. Wang, J. Perepezko, Inheritance from glass to liquid: *β* relaxation depresses the nucleation of crystals, Acta Materialia 185 (2020) 38-44.

[47] J. Qiao, Q. Wang, J. Pelletier, H. Kato, R. Casalini, D. Crespo, E. Pineda, Y. Yao, Y. Yang, Structural heterogeneities and mechanical behavior of amorphous alloys, Progress in Materials Science 104 (2019) 250-329.

[48] W.H. Wang, Dynamic relaxations and relaxation-property relationships in metallic glasses, Progress in Materials Science 106 (2019) 100561.

[49] Q. Yang, S.-X. Peng, Z. Wang, H.-B. Yu, Shadow glass transition as a thermodynamic signature of *β* relaxation in hyper-quenched metallic glasses, National Science Review 7(12) (2020) 1896-1905.

[50] R. Zhao, H. Jiang, P. Luo, L. Shen, P. Wen, Y. Sun, H. Bai, W. Wang, Reversible and irreversible *β*-relaxations in metallic glasses, Physical Review B 101(9) (2020) 094203.

[51] Q. Yang, C.-Q. Pei, H.-B. Yu, T. Feng, Metallic nanoglasses with promoted *β*-relaxation and tensile plasticity, Nano Letters 21(14) (2021) 6051-6056.

[52] Y. Duan, L. Zhang, J. Qiao, Y.-J. Wang, Y. Yang, T. Wada, H. Kato, J. Pelletier, E. Pineda, D. Crespo, Intrinsic correlation between the fraction of liquidlike zones and the *β* relaxation in high-entropy metallic glasses, Physical Review Letters 129(17) (2022) 175501.

[53] K. Ngai, Universal properties of relaxation and diffusion in complex materials: Originating from fundamental physics with rich applications, Progress in Materials Science 139 (2023) 101130.

[54] Z.-Y. Zhou, Q. Yang, H.-B. Yu, Toward atomic-scale understanding of structure-dynamics-properties relations for metallic glasses, Progress in Materials Science 145 (2024) 101311.

[55] L. Gao, H.-B. Yu, T.B. Schrøder, J.C. Dyre, Unified percolation scenario for the *α* and *β* processes in simple glass formers, Nature Physics 21(3) (2025) 471-479.
